# Plant-Derived Senotherapeutics for the Prevention and Treatment of Intervertebral Disc Degeneration and Aging

**DOI:** 10.3390/metabo14030146

**Published:** 2024-02-28

**Authors:** Eleni Mavrogonatou, Dimitris Kletsas

**Affiliations:** Laboratory of Cell Proliferation and Ageing, Institute of Biosciences and Applications, National Centre for Scientific Research “Demokritos”, 15341 Athens, Greece; dkletsas@bio.demokritos.gr

**Keywords:** senolytics, senomorphics, intervertebral disc, cellular senescence, plant-derived metabolites, extracellular matrix (ECM), senescence-associated secretory phenotype (SASP), low back pain

## Abstract

Chronic low back pain, a major cause of disability with a great global socioeconomic impact, has been inextricably associated with intervertebral disc degeneration. On the other hand, an enhanced number of senescent cells has been identified in aged and degenerated intervertebral discs and their senescence-associated secretory phenotype (SASP) has been connected with qualitative/quantitative alterations in the extracellular matrix and ultimately with the disturbance of tissue homeostasis. Given that selective elimination of senescent cells (by the so-called senolytics) or amendment of their secretome towards a less catabolic/inflammatory phenotype (by molecules known as senomorphics) has been reported to alleviate symptoms of several age-associated diseases and to improve tissue quality during aging, here we will review the emerging role of senolytic and senomorphic agents derived from plants and natural products against intervertebral disc degeneration. The mode of action of these senotherapeutics, as well as the challenges in their practical application, will also be explicitly discussed in an attempt to direct their more targeted and effective use in exclusive or combinatorial therapeutic schemes for the prevention and/or treatment of disc degenerative disorders.

## 1. Introduction

Overcoming premature mortality due to the evolution in medicine and healthcare, combined with the improvement of hygienic conditions, has led to an increased global life expectancy over the last century. However, an extension of the human lifespan is tightly linked with and considered to be among the greatest risk factors for the increased prevalence of chronic age-associated diseases because the irreversible process of aging is characterized by a progressive loss of physiological integrity. The population with an age over 65 years old (90% of which suffer from at least one chronic disease) is predicted to be approx. 1.6 billion worldwide by 2050, which is expected to impose a great socioeconomic burden [1,2]. In a comprehensive study on the assessment of incidence, prevalence and years lived with disability (YLDs) for 354 diseases and injuries from 1990 to 2017, low back pain (LBP)—experienced by more than 80% of individuals at least once in their lifetime—ranked first in YLDs [3,4].

LBP is one of the most common chronic age-associated pathological conditions, but it is also experienced by people of younger age, thus being a major public health problem with significant long-term consequences at both the individual and societal levels. Besides its impact on the quality of life, LBP results in a serious economic load, stemming from the direct costs of the healthcare system (including medications and care provided by medical doctors and practitioners) for the prevention and treatment of LBP-caused disability, as well as from the indirect costs due to activity limitation, decrease in productivity, absenteeism and early retirement from work [5]. The etiology of LBP is multifactorial; however, intervertebral disc degeneration has been yet indisputably considered a major contributor, if not its primary cause [6].

## 2. Intervertebral Disc—Intervertebral Disc Degeneration

Intervertebral discs (IVDs) lie between vertebrae and are responsible for providing the spinal column with a higher burden tolerance and higher flexibility in motion, as well as for the absorption of vibrations during standing, walking and carrying out daily activities. Each IVD comprises an outer annulus fibrosus (AF), an inner gelatinous nucleus pulposus (NP) and the cartilaginous endplates that segregate it from the inferior and superior vertebral body [7]. IVDs mainly consist of extracellular matrix (ECM) and are characterized by low cellularity. Nevertheless, these few embedded cells are the regulators of tissue homeostasis by producing structural ECM components and ECM-degrading enzymes. AF IVD cells are fibroblast-like, having an elongated morphology, parallelly aligned to the collagen fibers and are mainly type I collagen producers. On the other hand, NP IVD cells are similar to articular chondrocytes, expressing the same markers, i.e., type II collagen, aggrecan and SRY-Box Transcription Factor (SOX) 9 [8]. Maintenance of the subtle dynamic balance between synthesis and degradation of the IVD ECM is a prerequisite for the preservation of healthy tissue.

Low IVD cellularity is principally the outcome of the lack of vascularization in the tissue, resulting in limited disposal of nutrients that are almost exclusively mediated via diffusion through the cartilaginous endplate [9]. The absence of blood supply also leads to low oxygen concentrations and high lactic acid production as a result of the nearly mandatory glycolysis, as well as to the progressive accumulation of metabolic by-products, including reactive oxygen species (ROS). In addition, the negatively charged proteoglycans of the NP (with aggrecan being the main representative), by attracting cations from the extracellular microenvironment, increase osmolality to values higher than those prevailing in the majority of tissues, which become even higher in the daytime due to the mechanical loading-induced loss of hydration [10,11,12].

Intervertebral disc degeneration (IDD) is the pathophysiological condition of the progressive structural failure of the IVD that gradually allows the vascularization and innervation of the otherwise avascular and poorly innervated tissue, ultimately leading to chronic inflammation and pain [6]. IDD pathogenesis is a complicated process, the combined result of the stressful conditions prevailing in the IVD microenvironment mentioned above (nutrient deprivation, hypoxia, oxidative stress, mechanical stress, hyperosmotic stress), transient inflammatory responses, genetic predisposition and aging. With the progression of IDD, the equilibrium between ECM synthesis and degradation shifts towards catabolism, ensuing from the reduced production and deposition of ECM components on the one hand and the up-regulation of ECM-degrading enzymes, including matrix metalloproteinases (MMPs) and a disintegrin and metalloproteinase with thrombospondin motifs (ADAMTSs), on the other. Consequently, the AF ends up with decreased collagen content and loss of its organized fibrous network; the NP becomes more fibrotic due to proteoglycan loss, leading to water loss; and the cartilaginous endplate is calcified, worsening nutrient deficiency [13]. The enhanced and chronic inflammation of the degenerated IVD is evidenced by the increased secretion of pro-inflammatory cytokines [i.e., interleukin (IL)-1β and -6 and tumor necrosis factor (TNF)-α]. Macroscopically, degenerated IVDs present disruption of the lamellar structure of collagen fibers in the AF, loss of demarcation between the AF and the NP, fissures in both the AF and the NP, dehydration and reduced disc height [14], rendering X-ray imaging for the estimation of disc space narrowing, Pfirrmann grading system for Τ1ρ-, T2- and T2*-weighted magnetic resonance imaging (MRI) and histological scoring the most common methods for the qualitative evaluation of IDD [15,16,17].

## 3. Cellular Senescence Is a Main Etiologic Factor and/or Contributor in IDD Pathogenesis

As mentioned above, aging is a pivotal factor in IDD pathogenesis. On the other hand, cellular senescence is one of the hallmarks of aging [18]. The term cellular senescence was first introduced by Hayflick and Moorhead in the early 1960s to describe the permanent exit of human diploid embryonic lung fibroblasts from cell cycle progression after a finite number of cell divisions in vitro [19]. Besides this type of senescence—known as replicative senescence—which is the outcome of the telomere shortening occurring after repeated cell divisions, cells can also be driven to the so-called stress-induced premature senescence (SIPS) in response to various stressful conditions and extrinsic genotoxic stimuli [20]. Both the induction of replicative senescence and SIPS are related to the stimulation of a DNA damage response (DDR) in the cells through the activation of the cell cycle-regulating pathways p53/p21^WAF1^ and p16^INK4a^/pRB [21]. Apart from their inability to proliferate and the intracellular convergence of all senescence inducers into the DDR, other common features of all senescent cells are their flattened and irregular shape with numerous and enlarged vacuoles, their larger and deformed nucleus, cell membrane and cytoskeletal alterations, extensive macromolecular damage and accumulation of metabolic by-products, chromatin remodeling, mitochondrial dysfunction and enhanced lysosomal activity, all serving as markers for the identification and detection of senescent cells in vitro and in vivo. Most importantly, senescent cells present a specific secretome, mainly consisting of soluble chemokines, growth factors, pro-inflammatory mediators, bioactive lipids, MMPs and insoluble ECM components, the “senescence-associated secretory phenotype” (SASP). SASP is regulated by several signaling pathways [e.g., p53, p38 mitogen-activated protein kinase (MAPK), nuclear factor κB (NF-κB), cyclic GMP-AMP synthase/stimulator of interferon genes (cGAS/STING) and mammalian target of rapamycin (mTOR)] and is the mediator through which senescent cells exert most of their biological effects on adjacent cells and ECM [21,22,23]. Collectively, and based on all above-stated senescence-associated cellular modifications, to be firmly characterized as senescent, cells should assemble many of the following traits: overexpression of p16^INK4a^, p21^WAF1^ and p53, increased β-galactosidase activity (senescence-associated β-galactosidase, SA-β-Gal), accumulation of the by-product of oxidized proteins, lipids and metals (known as lipofuscin), accumulation of phosphorylated histone H2A.X and senescence-associated heterochromatin foci, down-regulation of lamin B1 or high mobility group box 1, increased oxidative load and altered expression of SASP factors towards a pro-inflammatory and catabolic phenotype [22].

By definition, and as opposed to the uncontrolled and indefinite ability of cancer cells to proliferate, cellular senescence may be considered an anticarcinogenic mechanism, while it also plays a beneficial role during normal development, tissue repair and against organ fibrosis [24]. Nevertheless, accumulation of senescent cells (e.g., due to organismal aging) may severely impair tissue microarchitecture via their SASP, substantially contributing to the manifestation or promotion of age-associated disorders [21,22]. After the first report on the existence of senescent cells in human IVDs by Roberts et al. [25], an enhanced number of senescent cells in aged and degenerated IVDs has been identified and confirmed [26], even very early in life [6]. Given the extremely low percentage of proliferating IVD cells in healthy tissues measured in vivo, senescence of IVD cells is most plausible to be SIPS as a response to the stresses prevailing in the tissue mentioned above (i.e., nutrient, hyperosmotic, hypoxic, mechanical and oxidative stress) rather than replicative [27]. In parallel, senescent cells’ accumulation may be accelerated in the particular tissue as their self-clearance by the immune system is attenuated due to the absence of vascularization [23]. Senescence-associated ECM changes in the IVD that could be causative factors of or contributors to ECM breakdown and the development of IDD have been investigated using several cell models and species in vitro and in vivo and include, amongst others: up-regulation of MMP-1, -2, -3, -7, -9, -10 and -13 and of ADAMTS-2, -4 and -5, down-regulation of type I and type II collagen, aggrecan and tissue inhibitor of metalloproteinase (TIMP)-1, -2, -3 and -4, suppression of the proteoglycans versican, decorin and biglycan, enhanced ADAMTS- and MMP-mediated aggrecan fragmentation, lower total glycosaminoglycan (GAG) content and down-regulation of SOX9 [21,22,28]. Thus, targeting senescent cells in the IVD seems a rational therapeutic alternative to pursue for IDD treatment, especially given the limitations of the hitherto employed curative strategies for LBP (ranging from the non-invasive administration of analgesics, exercise and physiotherapy to the invasive and often risky disc and spinal surgical procedures) that mostly target symptoms’ alleviation without addressing the causes of the disease [29,30]. Indeed, selective removal of p16^INK4a^-positive senescent cells in p16-3MR transgenic mice has been shown to ameliorate multiple age-associated changes within the IVD tissue [31], while p16^INK4a^ deletion has been reported to decrease ROS levels, the number of senescent cells and the SASP, as well as to rescue disc height index and ECM components’ expression levels in a mouse tail suspension-induced IDD model [32].

## 4. Senotherapeutics

The burst in the field of research for the development of therapies based on the selective targeting of senescent cells was triggered by the studies of Baker et al., in which the use of the transgene INK-ATTAC for inducible elimination of p16^INK4a^-positive senescent cells delayed the onset or attenuated the progression of already established age-related phenotypes in several tissues and extended median lifespan in mice [33,34]. On the other hand, transplantation of senescent cells in healthy tissues has been shown to promote degeneration, e.g., transplantation of senescent chondrocytes into the knee region of wild-type mice provided functional, radiographic and histological evidence for osteoarthritis (OA) induction [35]. Τhe consequent need for the attainment of improved resilience without genetic manipulation and its potential undesirable side-effects boosted the discovery of senolytics, that is, low-molecular-weight compounds that are selectively cytotoxic for senescent cells by targeting senescence-specific traits. First-generation senolytics (e.g., navitoclax, ABT-737, A-1331852, A-1155463) generally acted by interfering with the ability of senescent cells to resist apoptosis and were mainly inhibitors of the anti-apoptotic B-cell lymphoma 2 (Bcl-2) family proteins. Other reported senolytics targeted p53, p21^WAF1^, phosphoinositide 3-kinase (PI3K)/protein kinase B (Akt), serpins, ephrins, tyrosine kinases, hypoxia-inducible factor-1α (HIF-1α), forkhead box O (FOXO) 4, heat shock protein (HSP) 90 or were general cytotoxic agents that became senescence-specific through their encapsulation with β (1,4)-galacto-oligosaccharides by exploiting senescence-associated increased lysosomal β-galactosidase activity [2,36,37,38,39]. Apart from senolysis induction, eliciting senostasis (or senomorphism, referring to the suppression of specific senescent traits) is another means to counteract the unfavorable effects of senescence. Senomorphics are molecules that reduce the detrimental impact of senescent cells, mainly by blocking their SASP, thus conferring health benefits without removing senescent cells, contrastingly to the systemic application of senolytic drugs. Senomorphics are very often compounds with numerous biological activities and pleiotropic effects through the regulation of various signaling pathways, including those of NF-κB, PI3K/Akt, mTOR, IL-1α, p38 MAPK, nuclear factor erythroid 2-related factor 2 (Nrf2) and Janus kinase (JAK) [2,38]. Notably, senescent cells are highly heterogeneous, with their phenotype and secretome being dependent on the cell type/tissue, the context and the stimulus provoking senescence. Thus, it does not seem probable for the moment that a single senotherapeutic (the term used to describe both senolytics and senomorphics) would exert a universal anti-senescence action. Accordingly, a perpetual requirement for the discovery of novel and improved or the repurposing of already available active compounds with senolytic or senomorphic activity at the tissue and organ levels remains.

In this direction, numerous natural compounds have been recently discovered to be effective senotherapeutic agents and have been validated in animal models, which has rendered them promising candidates for current and future clinical applications [2]. In this review, we will focus on plant-derived compounds with a documented beneficial health effect against IDD. In the following parts, plant-derived compounds with a reported, as well as with a potential senotherapeutic activity in the IVD, will be presented. The mode of action (senomorphic and/or senolytic activity) of these senotherapeutics, the implicated molecular pathways through which their action is exerted and the challenges in their practical application to alleviate IDD will also be thoroughly discussed.

## 5. Plant-Derived Compounds with a Reported Senotherapeutic Activity in the IVD

Plant-derived metabolites are divided into two main categories: the primary metabolites—essential for survival, adaptation, growth, development and reproduction—and the secondary metabolites—which, even though not fatal when absent, are necessary for defense, resistance and long-term organismal maintenance [40]. Plant-derived secondary metabolites are subdivided into three classes: the terpenoids, the phenolic compounds (flavonoids) and the nitrogen-containing compounds (alkaloids), with terpenoids and polyphenols, such as anthocyanins, flavonols, isoflavones and chalcones, lying among the major bioactive compounds extracted from medicinal plants [40,41,42]. Due to their wide range of biological activities (anti-oxidant, anti-inflammatory, anti-cancer and anti-microbial) and their low toxicity for mammals, plant-derived secondary metabolites have found nutritional, cosmetic, agricultural and even pharmaceutical uses [40]. It is worth-mentioning that traditional medicine has a long history of using natural products for treating various diseases because of their abundance, high efficacy, low cost and minimal side effects compared with synthetic chemicals [43,44], while a great number of modern medicines have been developed on the basis of natural compounds [40]. Anti-oxidant effects of dietary phytochemicals are generally mediated via mitochondrial ROS levels’ down-regulation and increased superoxide dismutase (SOD) activity, whereas their anti-inflammatory activities stem from the inhibition of IL-1β, TNF-α, cyclooxygenase 2 (COX-2), inducible nitric oxide synthase (iNOS), prostaglandin E2 (PGE2) and IL-6 [13]. In addition to their other long-established bioactivities, natural dietary flavonoids, widely consumed in fruits and vegetables, have been recently shown to alleviate senescence in numerous cell types and organs, targeting diverse molecules regulating aging-related pathways. In detail, they have been reported to show senotherapeutic effects in fibroblasts, vascular smooth muscle cells, chondrocytes, synovial cells, keratinocytes, neural cells, hepatic cells and pre-adipocytes in vitro, as well as in various tissues and organs of aged mice and rats in vivo [39].

We will start by reviewing a number of plant-derived bioactive compounds with a reported senotherapeutic action in the IVD in vitro and in vivo (Table 1). Data on the protective role of these molecules in the cartilage will be presented as well, given the similarity of the NP IVD cells with articular chondrocytes.

### 5.1. Apigenin

**Figure 1:**
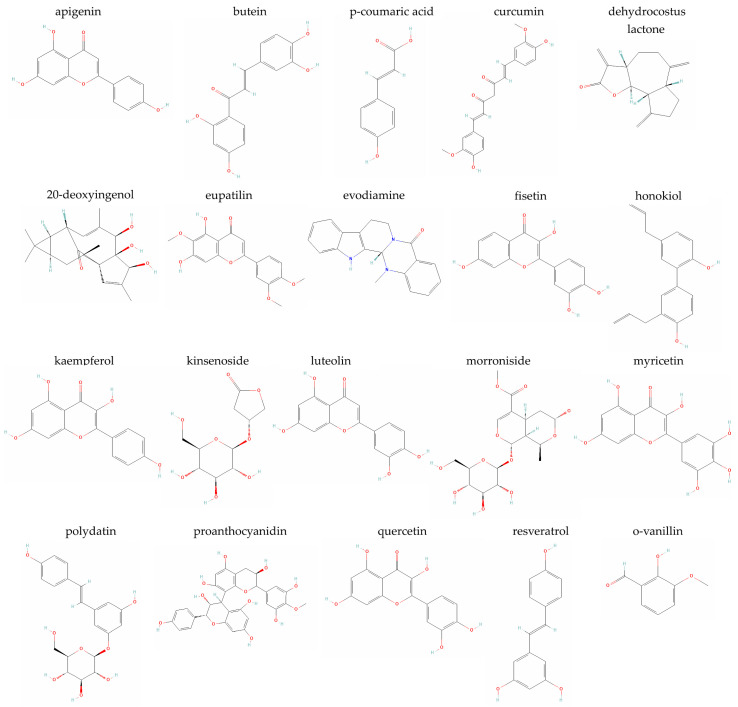
Chemical structures of plant-derived metabolites with a reported senotherapeutic activity in the intervertebral disc, retrieved from the public chemical database PubChem (https://pubchem.ncbi.nlm.nih.gov).

Apigenin (4′,5,7-trihydroxyflavone) is one of the most widely available natural flavonoids in the plant kingdom, met in large amounts in several vegetables (e.g., parsley, celery, onions, beans, broccoli), fruits (e.g., cherries, apples, grapes, oranges), herbs/herbal medicines and spices (e.g., chamomile, thyme, basil, cilantro and oregano), as well as plant-based beverages (e.g., tea, beer and wine) [45,75,76,77]. Even though the Asteraceae family (including *Achillea*, *Artemisia*, *Matricaria* and *Tanacetum* genera) is the main source of this compound, alternative forms or derivatives of apigenin have also been found in species of other plant families, such as the Fabaceae and the Lamiaceae [78]. Apigenin officially belongs to the flavone subclass of flavonoids (Figure 1) and is biosynthesized through the phenylpropanoid pathway from both phenylalanine and tyrosine [78]. It has been shown to possess anti-oxidant, anti-inflammatory, anti-aggregation and anti-mutagenic properties, which render it a very promising natural bioactive molecule with nutraceutical potential for the prevention and treatment of several inflammatory, autoimmune and degenerative diseases, including diabetes, neurocognitive, impairments, mood disorders, cancer, multiple sclerosis, viral infections, etc. [45,78,79,80,81] Apigenin has been reported to exert a senomorphic action through the inhibition of IκBζ expression in bleomycin-induced senescent normal human skin fibroblasts in vitro and in the kidneys of aged rats in vivo [79].

Given the beneficial effects previously reported for apigenin on pathologies closely related to the IVD tissues, i.e., against knee OA in humans [82], assessment of its putative protective role against IDD was a plausible research goal. In accordance with studies reporting that mTOR signaling is essential for maintaining IVD homeostasis and that autophagic flux disruption is implicated in cellular senescence and apoptosis during IDD development [83,84,85,86], apigenin has been shown to attenuate oxidative stress-induced senescence of NP IVD cells by increasing autophagy via the induction of the AMPK/mTOR/transcription factor EB (TFEB) signaling pathway [45]. In detail, apigenin treatment reversed the tert-butyl hydroperoxide (TBHP)-induced up-regulation of p62 expression and LC3-II/LC3-I ratio (thus inhibiting autophagosome-lysosome fusion interruption and lysosome dysfunction), the TBHP-induced p21^WAF1^ and p16^INK4a^ up-regulation and increased SA-β-Gal activity, as well as the TBHP-induced imbalance between ECM synthesis and breakdown (due to the decreased expression of type II collagen and proteoglycan and the increased expression of MMP-13 and ADAMTS-5) in rat NP IVD cells. Treatment with the known autophagic flux inhibitor chloroquine and siRNA-mediated TFEB knocking- down inhibited apigenin’s protective effects [45]. The protective effect of apigenin against IDD progression has also been confirmed in vivo using a needle puncture-induced IDD rat model: apigenin administration has been shown to restore to a degree p16^INK4a^, LC3-II and type II collagen expression levels, expression of the ECM-degrading enzymes MMP-1, MMP-2, MMP-3, MMP-9, ADAMTS-4 and ADAMTS-5, expression of cytokines and inflammatory mediators [e.g., IL-1β, IL-2, IL-6, IL-8 and IL-17, interferon (IFN)-γ, TNF-α], GAG and proteoglycan content, histological and Pfirrmann scores, as well as disc height of IDD specimens, suggesting an improvement of the tissue quality and a shift towards normal functioning [45,87].

### 5.2. Butein

Butein (2′,3,4,4′-tetrahydroxychalcone, mainly found in the stem bark of cashews and *Rhus verniciflua* Stokes) belongs to the chalcone subclass of flavonoids (Figure 1) and can be isolated from several plants of the Anacardiaceae, Asteraceae and Fabaceae families, including *Bidens bipinnata*, *Butea monosperma*, *Dahlia variabilis*, *Dalbergia odorifera*, *Millettia nitida*, *Semecarpus anacardium* and *Toxicodendron vernicifluum*, as well as other species of the *Dahlia*, *Butea*, *Searsia* and *Coreopsis* genera [44,88]. Butein and butein-containing crude plant extracts have long found a medicinal use due to the compound’s anti-oxidant, anti-inflammatory, anti-angiogenic, anti-cancer, anti-diabetic, anti-nephritic, hypotensive, neuroprotective and anti-bacterial properties [44,88,89,90]. Even though butein mainly acts as an IKKβ/NF-κB inhibitor, it has also been shown to modulate numerous other signaling pathways [44], which explains the broad spectrum of butein-treatable conditions reported so far. More specifically, butein has traditionally been used for curing cancer, inflammatory diseases, diabetes, atherosclerosis, hepatic disorders, ulcers, kidney diseases, eye diseases, rheumatoid arthritis, neuropathy, dementia, bleeding, cough, obesity, diarrhea, dysentery, liver fibrosis, tuberculosis, hypertension, infectious diseases, ischemia, malaria, paralysis, etc. [44,90] In the joint cartilage, butein significantly inhibited the IL-1β-induced production of nitric oxide (NO) and PGE2, expression of COX-2, iNOS, TNF-α, IL-6 and MMP-13, degradation of type II collagen and SOX9, as well as MMP-1, MMP-3, ADAMTS-4 and ADAMTS-5 gene expression in human OA chondrocytes in vitro by inactivating the NF-κB signaling pathway and it has been shown to reduce cartilage erosion and alleviate synovitis in a mouse OA model in vivo [91].

The incidence of apoptosis and senescence has been reported to increase as a response to hyperglycemia in rat NP cells in vitro, as well as in NP tissues from diabetic rats [92,93,94,95]. On the other hand, the expression of sirtuin (SIRT) 1—known to de-acetylate cell cycle-regulating transcription factors, such as p53, FOXO and NF-κΒ, being thus implicated in anti-apoptosis and anti-senescence mechanisms [96,97]—has been shown to decrease in degenerated NP tissues in rats and humans [68,98]. In addition to its anti-oxidant effect evidenced by the elimination of high glucose-induced intracellular ROS levels in rat NP IVD cells in vitro, butein has been shown to restrict high glucose-induced senescence by attenuating the increased percentage of SA-β-Gal-positive cells and p21^WAF1^ and p16^INK4a^ levels in the same cell model [46]. Anti-senescence effects of butein were hampered in cells pre-incubated with the SIRT1 inhibitor Ex527, ascribing to SIRT1 a key role in the described phenomena [46]. The protective effect of butein was validated by in vivo experiments using a streptozotocin-/puncture-induced rat model of diabetes and IDD [46]. More specifically, the proportion of p16^INK4a^-positive cells was found to decrease in the NP tissue sections of the butein-treated diabetic IDD rats compared with the diabetic IDD rats in the absence of butein, in parallel to the noticeable observed up-regulation of SIRT1 and down-regulation of acetylated p53. Moreover, butein administration secured the preservation of a better quality of the NP structure (as estimated by the higher histological scores of stained with safranin O/fast green and hematoxylin and eosin IVD specimens) and of higher T2-weighted MRI signal intensities in diabetic IDD rats.

### 5.3. p-Coumaric Acid

p-Coumaric acid (4-hydroxycinnamic acid) is a very abundant plant-derived secondary metabolite in nature, found in botanical families such as the Apiaceae, Compositae, Cucurbitaceae and Poaceae and genera including *Cynodon*, *Cucumis* and *Daucus* [99]. Its main dietary sources are a wide variety of edible plants and plant products, such as herbs (e.g., basil, garlic), fruits (e.g., apples, pears), vegetables (e.g., carrots, peas, potatoes, tomatoes), seeds and grains (e.g., peanuts, beans, rice), as well as fungi, cranberry syrups, grape juices and beverages (e.g., beer, wine, coffee, tea, chocolate) [100,101]. p-Coumaric acid (Figure 1) is a hydroxyl derivative of cinnamic acid and may be synthesized from both phenylalanine and tyrosine. From phenylalanine, cinnamic acid is formed by non-oxidative deamination and then, by oxidation at C-4, is converted to p-coumaric acid; from tyrosine, p-coumaric acid is formed directly by deamination [78]. In its turn, p-coumaric acid may transform into other secondary metabolites (e.g., into apigenin) [78,101]. The advantage of p-coumaric acid over other phenolic compounds lies in its higher bioavailability due to its ability to exist in a free or conjugated with other molecules form and due to its rapid metabolism and easy absorption in the intestine [100]. p-Coumaric acid has been shown to possess anti-oxidant, anti-inflammatory, anti-apoptotic, anti-platelet, anti-melanogenic, anti-neoplastic, cardioprotective, neuroprotective and anti-microbial properties, amongst others [101,102,103,104,105]. These properties may justify its pharmacological potential against numerous pathologies, including cancer, cardiovascular diseases, neuroinflammatory diseases, kidney diseases, liver diseases, diabetes and other metabolic disorders, as well as its large use in cosmetics, foods and pharmaceutical products [100,101]. p-Coumaric acid exerts its effects through the regulation of several signaling pathways, including the MAPK signaling pathway and the Bcl-2 antagonist X (Bax)/Bcl-2-p53 axis, the inhibition of NF-κΒ activation, the modulation of the cytoplasmic-to-nuclear ratio of Nrf2, the down-regulation of Toll-like receptor (TLR)-4 activation, the regulation of molecules belonging to the anti-oxidant cellular response [i.e., glutathione, malondialdehyde (MDA), SOD, catalase], while autophagy has emerged as a novel molecular mode of action of p-coumaric acid, introducing alternative therapeutic pathways for this compound [100,101].

Treatment with p-coumaric acid has been shown to effectively inhibit IL-1β-induced senescence in rat chondrocytes in vitro—as evidenced by the decrease of p16^INK4a^ protein expression and SA-β-Gal activity, the down-regulation of COX-2, iNOS, MMP-1, -3 and -13, ADAMTS-4 and -5 and the up-regulation of type II collagen and aggrecan—via inhibition of the MAPK and NF-κB signaling pathways [106]. The protective role of p-coumaric acid in cartilage has also been shown in vivo as it could alleviate the development of OA and rheumatoid arthritis in arthritis rat models [106,107,108,109]. In accordance with its chondroprotective role, p-coumaric acid has been found to mitigate H_2_O_2_-induced senescence traits in human NP IVD cells in vitro, as shown by the decrease of H_2_O_2_-induced SA-β-Gal-positive staining, the decline of H_2_O_2_-induced p16^INK4a^, p53, COX-2, iNOS gene expression, the reversal of H_2_O_2_-induced cell cycle arrest and the up-regulation of aggrecan and type II collagen expression [47]. In favor of the protective effect of p-coumaric acid on IVD cells, a subfraction of an acetone extract from Violina pumpkin (*Cucurbita moschata*) leaves—consisting almost entirely of p-coumaric acid—has been reported to result in the attenuation of H_2_O_2_-induced intracellular ROS levels, the overexpression of SOX9, transcriptional repressor GATA binding 1 (TRPS1) and FOXO3a, the up-regulation of aggrecan and type II collagen, along with the down-regulation of MMP-13 and the elevated expression levels of Nrf2, SIRT1 and SOD2 in degenerated human IVD cells [110].

### 5.4. Curcumin

Curcumin (diferuloylmethane), one of the most well-known plant-derived compounds, is an active natural, low molecular weight, lipophilic polyphenol (Figure 1) that is mainly extracted from the rhizome of the perennial herbaceous flowering plant Turmeric (*Curcuma longa*) of the Zingiberaceae family, cultivated in Asian countries [111,112]. Curcumin has been shown to possess anti-oxidant, anti-inflammatory, anti-aging, anti-tumor, anti-microbial, neurotrophic and context- and cell type-dependent pro-apoptotic or anti-apoptotic properties, while it regulates cell proliferation-, autophagy- and senescence-associated signaling pathways [13,48,113,114,115,116,117,118]. Due to its widespread biological activities, traditionally, it has been extensively used for the prevention/treatment of several age-associated pathological conditions, including cancer, atherosclerosis, cardiovascular diseases, diabetes, hypertension, neurodegenerative diseases, rheumatoid arthritis, osteoporosis, kidney diseases, hepatic disorders, chronic inflammations and infectious diseases, sinusitis and optical disorders [48,111,114,115,116,119]. Furthermore, the anti-inflammatory effects of curcumin on injury-related skin infections, as well as on injuries of the cartilaginous tissue, tendons and bone, have also been reported [120].

Curcumin has been shown to alleviate IDD by up-regulating autophagy, inhibiting apoptosis and confining phenotype loss of cartilaginous endplate cells induced by high-intensity cyclic tension [121]. It has also been reported to reduce levels of MMP-1, -3 and -13 and of IL-1β, -6 and -8 in IL-1β-human IVD cells in an NF-κB-independent manner by activating p38 and extracellular signal-regulated kinase (ERK) MAPKs, down-regulating TLR2 expression and inhibiting c-Jun N-terminal kinase (JNK) [115]. A curcumin-mediated suppressive effect on the NF-κB pathway has also been shown in a rat lumbar IDD model [122]. Specifically regarding its senotherapeutic potential in the IVD, curcumin has been shown to mitigate the IL-1β-induced increase in the percentage of SA-β-Gal-positive human NP IVD cells and the IL-1β-induced enhanced MMP-2 and -3 expression in the supernatants of human NP IVD cells [49]. Curcumin treatment reversed the TBHP-induced increase in p16^INK4a^ protein levels and in SA-β-Gal activity and the TBHP-induced decrease in EdU staining, restored type II collagen, aggrecan, MMP-3, MMP-13, ADAMTS-4 and ADAMTS-5 mRNA and protein levels and eliminated ROS intracellular levels in human NP IVD cells [48]. In addition, it re-established mitochondrial function, as demonstrated by the restoration of mitochondrial membrane potential, intracellular MDA levels, SOD activity, ATP content and mitochondrial permeability transition pore (mPTP) opening. Protective effects were mediated through the curcumin-induced annulment of TBHP-provoked interruption of autophagosome-lysosome fusion and impairment of lysosomal function in an AMP-activated protein kinase (AMPK)/mTOR/Unc-51-like kinase (ULK) 1-dependent manner [48]. In a puncture-induced IDD rat model, in vivo curcumin administration has been shown to result in higher T2-weighted signal intensities and lower Pfirrmann MRI grade scores, as well as in the restoration of disc height and disc structure compared to the IDD group. Furthermore, curcumin treatment increased the levels of type II collagen and aggrecan and the ratio of LC3-II/I and decreased the levels of cleaved caspase-3, MMP-13, ADAMTS-4, ADAMTS-5, p62 and H_2_O_2_ content compared with the IDD group [48]. Treatment with curcumin significantly suppressed IL-1β and IL-6 levels, significantly reduced iNOS and MMP-9 levels and significantly decreased COX-2 and transforming growth factor (TGF)-β1/2 mRNA levels in rats with surgically-induced lumbar IDD compared with the untreated group [123]. In their comprehensive study, Cherif et al. have explicitly attributed a senolytic effect to curcumin on human AF and NP IVD cells exerted via the suppression of the JNK pathway along with a reduction in the secretion of SASP factors through the down-regulation of the Nrf2 and NF-kB pathways [50]. In detail, curcumin treatment led to a significant decrease in senescent cells (as revealed by the lower p16^INK4a^ staining), a rise in apoptosis (as revealed by the increased number of caspase-3-positive cells) and a significant increase in the number of Ki-67-positive (proliferating) cells in monolayer cultures of NP cells isolated from human degenerate IVDs. The specificity of the pro-apoptotic effect of curcumin against senescent cells was evidenced by double-staining experiments revealing the presence of p16^INK4a^(+)/Ki-67(-) and p16^INK4a^(+)/caspase-3(+) cells in the samples. Moreover, curcumin increased metabolic activity, caspase 3/7 activity and apoptosis selectively in cells from degenerate IVDs and not in cells from non-mildly-degenerate IVDs. In addition, curcumin resulted in increased proteoglycan content and type II collagen levels and decreased levels of the SASP inflammatory cytokines IL-6 and -8 and the ECM-degrading enzymes MMP-3 and -13. The protective effect of curcumin was confirmed using a pellet culture, better mimicking the three-dimensional in vivo conditions of the tissue [50].

### 5.5. Dehydrocostus Lactone

Dehydrocostus lactone (DHE) is a natural sesquiterpene lactone (Figure 1) isolated from medicinal plants (such as *Inula helenium* L. of the Asteraceae family and *Saussurea lappa* Clarke of the Compositae family) that has anti-oxidant, anti-inflammatory, anti-tumor, anti-ulcer, hepatoprotective and immunomodulatory properties [51,124,125,126,127]. Its biological activities are mainly exerted through the modulation of NF-κB, MAPK and PI3K/Akt signaling pathways [125,126,127,128,129]. It has been reported that DHE significantly suppressed glioma development, lipopolysaccharide-induced acute lung injury and macrophage activation, digestive tract diseases, as well as attenuated osteoclastogenesis and osteoclast-induced bone loss [51,125,128,129].

Interestingly, DHE treatment partially attenuated TNF-α-induced senescence in rat NP IVD cells, as demonstrated by the decrease in the percentage of SA-β-Gal-positive cells and the down-regulation of p53 and p21^WAF1^ [51]. This anti-senescence effect was accompanied by the reversal to a degree of the TNF-α-induced catabolic phenotype of the cells (characterized by the up-regulation of MMP-3, -7, -9 and -13 and the decrease of type II collagen and aggrecan levels) and the prevention of the loss of proteoglycans, as shown by alcian blue staining. DHE inhibited TNF-α-induced activation of NF-κB and MAPK (p38, JNK and ERK) signaling pathways in IVD cells and prevented dsDNA release-induced STING-TANK-binding kinase 1 (TBK1) activation. Consistent with the in vitro findings, intraperitoneal injection of DHE in a spinal instability-induced mouse model in vivo partly ameliorated the loss of disc height, significantly restored the quality of the disc structure, increased histological scores and alleviated the expression of TNF-α, IL-1β and the breakdown of aggrecan in the lumbar disc tissue, all indicative of a protective role of DHE against IDD progression [51].

### 5.6. 20-Deoxyingenol

20-Deoxyingenol is a diterpene isolated from the seeds of *Euphorbia lathyris* L., a biennial herb native to the Mediterranean area that belongs to the Euphorbiaceae family [130]. Due to their high content in lathyrane diterpenes, seeds of *E. lathyris* L. have long been used as a traditional medicine but could prove to be promising for use in modern medicine, as well [130].

20-Deoxyingenol has already been reported to alleviate OA by activating TFEB in chondrocytes [131]. On the other hand, nuclear localization of TFEB has been found to decline in TBHP-treated NP IVD cells, as well as in degenerated rat NP tissue. Accordingly, TFEB overexpression restored the TBHP-induced autophagic flux disruption and defended NP cells against apoptosis and senescence, a protective effect that was attenuated by chloroquine-mediated autophagy inhibition. In vitro findings were validated by the amelioration of IDD development in a rat puncture-induced IDD model after TFEB overexpression [85]. Based on the aforementioned data, it has been recently shown that 20-deoxyingenol treatment decreased TBHP-induced cGAS, STING, p53, p21^WAF1^, p16^INK4a^ overexpression and SA-β-Gal activity in rat NP cells [52]. Moreover, TBHP and 20-deoxyingenol co-treatment resulted in a partial restoration of the autophagic flux. Notably, senescence traits were eliminated by the autophagy inhibitor bafilomycin A1, which can block autophagosome-lysosome fusion and inhibit acidification and protein degradation. Additionally, the TFEB-mediated anti-senescence mode of action of 20-deoxyingenol through the autophagy-lysosome pathway was confirmed using a TFEB shRNA. Most importantly, all in vitro findings were confirmed by in vivo experiments in a rat IDD model, as demonstrated by disc height index calculations based on X-ray images, Pfirrmann grading based on MRI images, histological scores, immunohistochemical and tissue immunofluorescence analysis, showing that 20-deoxyingenol effectively hindered IDD progression via TFEB [52].

### 5.7. Eupatilin

Eupatilin (5,7-dihydroxy-3′,4′,6-trimethoxyflavone) (Figure 1) is the primary flavonoid extracted from the herbaceous perennial plant *Artemisia argyi* of the Asteraceae family with pharmacological potential due to its anti-cancer, anti-oxidant and anti-inflammatory properties [132]. Eupatilin’s biological activities have been shown to be mediated by the regulation of several signaling pathways, including NF-κB, MAPK, Nrf2 and JAK2/signal transducer and activator of transcription (STAT) 3, through which the compound has been reported to suppress allergic inflammatory responses, lung injury in sepsis and ovalbumin-induced asthma [133,134,135,136]. Furthermore, eupatilin has been reported to exert chondroprotective effects in IL-1β-stimulated human OA chondrocytes in vitro and antinociceptive and chondroprotective properties in a rat model of OA in vivo by downregulating phosphorylated levels of JNK, oxidative damage and catabolic activity [137].

Regarding its senotherapeutic potential in the IVD, eupatilin treatment has been shown to attenuate TNF-α-induced senescence in rat NP IVD cells in vitro by reducing the number of SA-β-Gal-positive cells and the TNF-α-enhanced p21^WAF1^ and p53 protein expression levels [53]. In the same study, eupatilin significantly inhibited TNF-α-induced inflammatory response and ECM degradation by partially reversing the up-regulation of TNF-α, IL-1β, MMP-3, -7, -9 and -13 and the down-regulation of SOX9 and type II collagen, as well as the TNF-α-induced loss of proteoglycans, as shown by alcian blue staining [53]. NF-κB and MAPK signaling were found to be implicated in the protective role exerted by eupatilin against ECM degradation and cellular senescence of rat NP IVD cells because eupatilin resulted in the decrease of p65 phosphorylation and translocation from the cytoplasm to the nucleus, IκBα phosphorylation and degradation and p38, JNK, and ERK activation. In favor of the in vitro findings, eupatilin intravenous injection was demonstrated to ameliorate the puncture-induced caudal IDD in a rat model, based on the improved X-ray, MRI and disc height index data, on the staining of IVD sections with safranin O/fast green and hematoxylin and eosin and on immunofluorescence staining for type II collagen, all supporting reduced destruction of the disc tissue and structure [53].

### 5.8. Evodiamine

Evodiamine is an indole quinazoline alkaloid (Figure 1) extracted from the dried small berry fruit of *Evodia rutaecarpa* of the Rutaceae plant family [138]. It possesses anti-oxidant, anti-apoptosis, anti-inflammatory, anti-tumor, anti-infection, anti-ulcer, anti-vomiting, analgesic and neuroprotective properties, explaining its wide pharmacological use to treat various diseases, such as diarrhea, ulcerative colitis, beriberi, liver pathologies, depressive disorders, etc. [138,139,140,141] Evodiamine may act through the regulation of several molecular pathways; for example, it has been reported to reduce the peripheral hypersensitivity and anxiety of mice with nerve injury or inflammation through transient receptor potential vanilloid (TRPV) 1 [142], to exert a protective effect on lipopolysaccharide (LPS)-treated rat kidney cells in vitro and LPS-induced acute kidney injury and cytotoxicity in rats in vivo through the regulation of ROS/NF-κΒ-mediated inflammation [143] and to act as an anti-cancer molecule via SIRT1 regulation [144].

This natural compound has been previously shown to possibly possess a protective potential against IDD by up-regulating SIRT1 and then activating the PI3K/Akt pathway to inhibit LPS-induced apoptosis, ECM degradation and inflammation in immortalized human NP IVD cells [144]. In a recent article, the authors showed that evodiamine treatment ameliorates the progression of IDD by alleviating mitochondrial dysfunctions (as shown by the decrease of mitochondria-derived ROS and the restoration of the mitochondrial membrane potential), ECM degradation (as shown by the reversal of MMP-3, MMP-13 and ADAMTS-4 up-regulation and of type II collagen and aggrecan down-regulation) and inflammation (as shown by the down-regulation of iNOS, COX-2, TNF-α, IL-1β and IL-6) via the Nrf2/heme oxygenase (HO)-1 and MAPK pathways using an in vitro model of TBHP-stimulated rat NP IVD cells, as well as an in vivo puncture-induced rat IDD model [54]. Notably, even though the authors mentioned that they used TBHP to provoke oxidative stress and senescence in their experimental models, implying the presentation of data on the effect of evodiamine on TBHP-induced up-regulation of senescence markers, in fact, induction of senescence, as well as the putative anti-senescence role of evodiamine, were not directly assessed in the particular study and thus remain to be investigated.

### 5.9. Fisetin

Fisetin (3,3′,4′,7-tetrahydroxyflavone) is a natural compound commonly found in a wide variety of plants belonging to the Fabaceae and Anacardiaceae families and is present in many fruits and vegetables, such as strawberries, apples, persimmons, grapes, kiwis, cucumbers and onions [145,146]. Chemical structure-wise, fisetin belongs to the flavonol subgroup of flavonoid polyphenols (Figure 1), along with kaempferol, myricetin and quercetin [146]. It has been shown to possess anti-oxidant, anti-inflammatory, anti-cancer, anti-diabetic, anti-bacterial, anti-viral and neuroprotective biological activities [2,146], mediated by several mechanisms of action on numerous molecular targets and signaling pathways, including Bcl-2, PI3K/Akt, ERK, JNK, aurora B kinase, p53, NF-κB, MMPs and mTOR [2,146,147], with no adverse effects reported, even when administered at high doses [148]. Fisetin is among the most studied natural compounds with an established senotherapeutic role [149,150], most likely exerted through the PI3K/Akt/mTOR and NF-κB molecular pathways [151]. It has been shown to reduce senescence markers in multiple murine and human tissues in a cell type-specific manner, to restore tissue homeostasis, to attenuate age-associated pathologies and to extend lifespan after its administration in progeroid and old mice [152]. Regarding its beneficial effects against pathological conditions of the musculoskeletal system, fisetin has been shown to counteract osteoporosis in in vitro and in vivo preclinical studies across animal species [153], to inhibit IL-1β-induced inflammatory response in human OA chondrocytes through activating SIRT1 and to attenuate the progression of OA in mice [154], as well as to ameliorate bone degeneration in the *Zmpste24*^−/−^ progeria murine model for Hutchinson–Gilford progeria syndrome [155].

In the IVD, fisetin has been reported to attenuate H_2_O_2_-induced apoptosis, inflammation (by decreasing elevated IL-6 and TNF-α expression and secretion) and ECM degradation (by restoring aggrecan, type II collagen and MMP-3 and -13 levels) in primary rat NP mesenchymal stem cells via SIRT1 [156]. Furthermore, in a recent study, fisetin has been reported to result in the Nrf2-mediated mitigation of oxidative stress-induced ferroptosis, in the attenuation of oxidative stress-induced senescence (by significantly decreasing the senescence marker p16^INK4a^ and reducing positive SA β-Gal staining) and in the reversal of the oxidative stress-induced catabolic phenotype (by reducing MMP-13 expression and elevating type II collagen levels) of TBHP-treated rat NP IVD cells in vitro [43]. These protective effects of fisetin were further validated in vivo in a rat model of puncture-induced IDD, in which fisetin administration restored loss of disc height and intensity, ameliorated tissue structure by inhibiting ECM degradation, limited lipid peroxidation and senescence by reducing glutathione peroxidase (GPX) 4 and p16^INK4a^ expression, respectively and increased expression levels of the Nrf2/HO-1 axis [43].

### 5.10. Honokiol

Honokiol (5,3′-Diallyl-2,4′-dihydroxybiphenyl) is a naturally occurring pleiotropic small-molecular weight polyphenol (Figure 1), mainly isolated from the leaves, roots and bark of plants belonging to the *Magnolia* genus and more specifically of *Magnolia grandiflora*, *Magnolia officinalis* and *Magnolia dealbata* [157,158,159,160]. Honokiol has been shown to possess anti-oxidant, anti-inflammatory, anti-apoptosis, anti-cancer and anti-aging properties and has been implicated in the maintenance of a normal mitochondrial function and autophagic flux [13,157,161]. Only a few of the signaling molecules and pathways through which honokiol exerts its protective effects are epidermal growth factor receptor (EGFR), STAT3, AMPK, Akt/mTOR, MAPKs, NF-kB, Nrf2 and SIRT3 [157]. Due to its extensive reported pharmaceutical potential and wide spectrum of therapeutic actions (e.g., anti-diabetic, anti-depressant, anti-microbial, anti-tumorigenic, anti-thrombotic, anti-hypertensive, anti-spasmodic, analgesic, anxiolytic, neuroprotective, cardioprotective, hepatoprotective) [157,158,159] combined with its low toxicity [160], honokiol has been traditionally used in the treatment of various diseases without any notable side-effects, including myocardial ischemia/reperfusion injury, pressure overload-induced cardiac hypertrophy, doxorubicin-induced cardiomyopathy, diabetes mellitus, reproductive disorders and inflammatory arthritis [157,161]. Interestingly, honokiol has been shown to restore TIMP-2 expression and to suppress senescence—as shown by the reduction of SA-β-Gal activity—in cigarette smoke-stimulated primary human foreskin fibroblasts [162] and to antagonize doxorubicin-induced senescence in cardiomyocytes [161].

Honokiol has been shown to exert a chondroprotective effect by suppressing IL-1β-induced iNOS and COX-2 overexpression, NO, PGE2, IL-6 and MMP-13 overproduction and type II collagen reduction via the regulation of the IKK/IκBα/NF-κB signaling pathway [163]. In the IVD, honokiol has been shown to inhibit H_2_O_2_-induced elevated ROS and MDA, overexpression of IL-6, COX-2, iNOS, MMP-3, MMP-13, ADAMTS-4 and ADAMTS-5 and down-regulation of type II collagen and SOX9 in rat NP cells in vitro via the suppression of the activation of thioredoxin-interacting protein (TXNIP)-NOD-like receptor protein 3 (NLRP3) inflammasome signal pathway and to ameliorate IDD in a puncture-induced IDD rat model [164]. Most importantly, honokiol treatment has been shown to restore redox status, mitochondrial dynamics, mitophagy and to reduce the increased percentage of SA-β-Gal-positive cells and overexpression of p16^INKa^ in TBHP-stimulated rat NP IVD cells in vitro by activating the AMPK- peroxisome proliferator-activated receptor gamma coactivator-1α (PGC-1α)-SIRT3 signaling pathway, while it ameliorated puncture-induced IDD in rats [55].

### 5.11. Kaempferol

Kaempferol [3,5,7-trihydroxy-2-(4-hydroxyphenyl)-4H-1-benzopyran-4-one] is a natural flavonol-type flavonoid, often isolated from tea, as well as numerous vegetables and fruits, including apples, beans, broccoli, brussel sprouts, cabbage, citrus fruits, gooseberries, grapefruit, grapes, kale, strawberries and tomatoes [165]. Kaempferol and its derivatives have been identified in many botanical families and in several medicinal plants, including *Aloe vera* (L.), *Delile*, *Euphorbia pekinensis* Rupr., *Ginkgo biloba* L. and *Rosmarinus officinalis*. It has a diphenylpropane structure (Figure 1) and is synthesized by condensation of 4-coumaroyl-CoA with three molecules of malonyl-CoA (with naringenin chalcone, naringenin and dihydrokaempferol as intermediates) and shows structural similarity with the hormone estrogen [165]. Kaempferol possesses many biological activities, such as anti-oxidant, anti-cancer, anti-inflammatory, anti-microbial, anti-platelet and anti-thrombotic, anti-diabetic, anti-allergic, anti-asthmatic, estrogenic, bone anabolic, analgesic and neuroprotective, mainly mediated by the NF-kB, MAPK and PI3K/Akt signaling pathways [165,166,167]. Consequently, it has been reported to have beneficial health effects against cancer, hypertension, vascular endothelial inflammation, cardiovascular diseases, abdominal pain, headaches, rheumatism, post-menopausal bone loss, colitis, liver and metabolic diseases, atherosclerosis, obesity, fibrotic disorders and acute lung injury. Especially regarding the musculoskeletal system, kaempferol has been reported to inhibit osteoclastogenesis [56], induce chondrogenesis [168] and prevent or delay OA through inhibition of the NF-κB signaling pathway [166]. Moreover, it has been shown to act as a senomorphic agent, given its inhibitory effect on SASP production in bleomycin-induced senescent skin fibroblasts [79].

Kaempferol—identified as the main active compound of the traditional Chinese medicine Du Zhong through network pharmacology-based integrative bioinformatics analysis—has been reported to suppress to a degree IL-1β-induced senescence of human NP IVD cells, as shown by the attenuation of the senescence markers p16^INK4a^, p21^WAF1^ and SA-β-Gal staining. In addition, kaempferol reinstated a functional anti-oxidative response by enhancing Nrf2, HO-1, NADPH-quinone oxidoreductase 1 (NQO-1), SOD1 and SOD2 expression and restored intracellular ROS levels, while IL-1β-induced alterations in ECM production- and degradation-related molecules (e.g., aggrecan, type II collagen, SOX9, fibronectin, MMP-3, MMP-13, ADAMTS-4 and ADAMTS-5) were also partially abolished by kaempferol treatment. Finally, kaempferol administration significantly relieved the IL-1β-stimulated increase in p38, JNK and ERK1/2 phosphorylation [56]. In favor of the IVD-protective role of kaempferol, the compound was found to be among the active ingredients of ZhiKe GanCao Decoction (ZKGCD)—a commonly used traditional Chinese medicine in the clinical treatment of IDD—based on network pharmacology, molecular docking and enrichment analysis [57].

### 5.12. Kinsenoside

Kinsenoside [3-(R)-3-β-D-glucopyranosyloxybutanolide] is a glycoside (Figure 1) considered to be the major bioactive compound of *Anoectochilus roxburghii*, a member of the Orchidaceae family, typically distributed throughout tropical and subtropical regions of Asia [58,169]. It has been shown to possess numerous properties with pharmacological potentials, such as anti-inflammatory, anti-oxidant, anti-apoptosis, anti-hyperglycemic, anti-hyperliposis, hepatoprotective and angioprotective [169,170,171,172] and has thus been commonly used in the past to treat diabetes, hyperlipemia, hyperglycemia, hepatitis, cough-variant asthma, osteoporosis, gouty arthritis and rheumatoid arthritis [169,173,174,175,176,177]. MAPK, NF-κΒ and vascular endothelial growth factor (VEGF) signaling pathways have been reported to be involved in the manifestation of kinsenoside’s biological activities [172,174].

It has been reported that kinsenoside mitigates TBHP-induced SA-β-Gal activity and p16^INK4a^ overexpression in rat NP IVD cells in vitro and results in their increased proliferative capacity [58]. In addition, mitochondrial ROS and loss of mitochondrial membrane potential that was markedly increased in TBHP-stimulated rat NP cells were demonstrated to be prevented by the administration of kinsenoside. shRNA-mediated Nrf2 knock-down compromised the protection of TBHP-treated NP cells by kinsenoside, pinpointing a central role of this transcription factor in kinsenoside-conferred relief in the IVD. In vitro findings were validated by in vivo experiments in a rat caudal puncture-induced IDD model, in which kinsenoside administration was shown to induce Nrf2 up-regulation and p16^INK4a^ down-regulation, to increase MRI signal intensities and histological scores and to decrease Pfirrmann grades in comparison with the IDD group, revealing a partial abolishment of disc degeneration and tissue destruction [58].

### 5.13. Luteolin

Luteolin (3′,4′,5,7-tetrahydroxy flavone) belongs to the flavone group of flavonoids and, along with its glycosides, has been isolated from species of the Asteraceae, Lamiaceae, Poaceae, Leguminosae and Scrophulariaceae plant families. Luteolin is widely distributed in vegetables, fruits, flowers, herbs and spices, such as carrots, peppers, cabbages, celery, parsley, broccoli, onion leaves, apple skins and chrysanthemum and has been traditionally used to treat hypertension, inflammatory diseases and cancer [59,178,179]. It is biosynthesized in the phenylpropanoid and flavonoid pathways, with naringenin being the key intermediate compound in its biosynthesis, and it has weak aqueous soluble properties [179]. Luteolin possesses two benzene rings, a third oxygen-containing ring, hydroxyl groups at carbons 5, 7, 3′ and 4′ positions and a 2–3 carbon double bond (Figure 1), the latter being associated with its main biochemical and biological activities [178]. Among its multiple biological activities lie anti-inflammatory, anti-cancer, anti-diabetic, anti-microbial, context-dependent pro- or anti-oxidant, cell cycle-regulating, estrogenic-regulating and neuroprotective activities, exerted through the modulation of numerous signaling molecules and pathways, including epidermal growth factor (EGF), VEGF, insulin-like growth factor-I (IGF-I), TLRs, NF-κB, MAPKs, PI3K/Akt, STAT3 and SIRT6 [59,178,179,180].

Luteolin has been shown to inhibit NF-κΒ activation and IL-1β-induced inflammation in rat chondrocytes in vitro and to attenuate OA progression in a rat model in vivo by inhibiting cartilage destruction and enhancing type II collagen expression [181]. In the IVD, luteolin has been reported to abrogate TNF-α-induced decrease in SIRT6 up-regulation and NF-κΒ activation in immortalized human NP IVD cells. Moreover, it has been shown to diminish TNF-α-induced inflammation and senescence by reversing IL-1β and IL-6 up-regulation and SA-β-Gal activity, p16^INK4a^, p21^WAF1^ and p53 overexpression, all partially annulled by siRNA-mediated SIRT6 knocking-down [59]. In accordance, SIRT6 overexpression has been shown to prevent matrix degradation through inhibition of the NF-κB pathway, to suppress senescence and apoptosis of NP cells by inducing autophagy and to ameliorate IDD in a puncture-induced rat model [181,182].

### 5.14. Morroniside

Morroniside is an iridoid glycoside (Figure 1) and the main active ingredient isolated from *Cornus officinalis* of the dogwood family Cornaceae [183]. Morroniside has been shown to exert many biological effects, such as anti-inflammatory, anti-apoptotic, anti-oxidant, cardioprotective and neuroprotective via several mechanisms and signaling pathways, i.e., STAT3, NF-κB, MAPK and PI3K/Akt pathways and molecules regulating the autophagic flux [184,185,186,187,188,189]. It has thus long been used to treat degenerative diseases of the nervous system, to inhibit platelet aggregation, to prevent diabetic angiopathies and renal damage, to treat OA and to reduce bone resorption [190].

Morroniside has been previously shown to attenuate apoptosis and pyroptosis of chondrocytes and to ameliorate OA development by inhibiting NF-κB signaling [190]. Furthermore, the morroniside-containing traditional medicine Liuwei Dihuang Decoction has been proven to be able to impede IDD progression via network pharmacology analysis combined with experimental validation [191]. In a recent study, morroniside has been demonstrated to down-regulate p53 expression and transcriptional activity, p21^WAF1^ mRNA and protein levels and to cause a significant decrease in the percentage of SA-β-Gal-positive H_2_O_2_-treated rat NP cells in vitro [60]. Additionally, it disrupted H_2_O_2_-induced phosphorylation of mammalian STE20-like protein kinase 1/2 (Mst1/2) and large tumor suppressor 1/2 (Lats1/2), reversed H_2_O_2_-induced reduction of Yes-associated protein (Yap) and transcriptional coactivator with PDZ-binding motif (Taz) and diminished H_2_O_2_-induced ROS increase [60]. The anti-senescence effect was abolished after the blockade of Hippo signaling by Yap/Taz inhibition, consistent with the established close association of Hippo signaling activity with NP cell senescence [192,193]. In vivo, morroniside has been shown to decrease to a great extent the elevated expression of p53 and p21^WAF1^ and positive SA-β-Gal staining, while it significantly alleviated the decrease in aggrecan and increase in ADAMTS-5 in the NP tissues of a lumbar spine instability surgery-induced mouse IDD model. Furthermore, morroniside restored histological scores and blocked structural and compositional impairments of the tissue, indicative of restraint in IDD progression [60].

### 5.15. Myricetin

Myricetin (3,3′,4′,5,5′,7-hexahydroxyflavone) is a member of the flavonol group of flavonoids, first isolated in the late eighteenth century from the bark of *Myrica nagi* Thunb. and is produced by several species of the Myricaceae, Anacardiaceae, Polygonaceae, Pinaceae and Primulaceae families [194,195]. It is widely found in fruits, vegetables, berries and blueberry leaves, nuts, rose petals, sea buckthorn, chia seeds, the Japanese raisin tree, carob extract, grape seed extract, garlic and beverages, such as tea and red wine [194,195,196]. Due to its structural similarity with quercetin (Figure 1), myricetin is also known as hydroxyquercetin [195]. Myricetin is mostly soluble in organic solvents and poorly soluble in water and aqueous buffers [194,195]. In the chemical structure of myricetin, flavone is substituted by hydroxyl groups at the 3, 3′, 4′, 5, 5′ and 7 positions, three of which form a pyrogallol group in the B-ring that provides myricetin with high anti-oxidant potential and other biological activities [197]. Consequently, this plant-derived flavonoid has been accepted for its nutraceutical potential based on its anti-oxidant, anti-inflammatory, anti-epileptic, anti-viral, anti-bacterial, anti-photoaging, neuroprotective, cardioprotective, hepatoprotective, gastroprotective and analgesic effects mediated by signaling molecules including NF-κB, Nrf2, PI3K/Akt, mTOR and STAT [194,195,196,198]. Myricetin shows great therapeutic potential against cancer, diabetes, obesity, liver injury, cardiovascular diseases and osteoporosis [195].

Myricetin has been reported to exert a protective effect on IL-1β-stimulated human chondrocytes in vitro and on a destabilization of the medial meniscus (DMM)-induced OA mouse model through the PI3K/Akt-mediated Nrf2/HO-1 signaling pathway [199]. Myricetin has been reported to reduce to a degree the percentage of SA-β-Gal-positive cells induced by H_2_O_2_ in human NP IVD cells, the levels of p16^INK4a^ and p21^WAF1^ and the levels of the pro-inflammatory SASP factors IL-6 and IL-8 in vitro [61]. Silencing of SERPINE1 (selected after RNA-seq, bioinformatics and gene ontology enrichment analysis) inhibited H_2_O_2_-induced cellular senescence, while SERPINE1 overexpression using an overexpression plasmid reversed the myricetin-induced anti-senescence and anti-inflammatory effects [61]. Furthermore, myricetin has been shown to exhibit a protective effect against H_2_O_2_-induced mitochondrial dysfunction (i.e., it moderated increased mitochondrial ROS and damaged mitochondrial membrane potential) and to prevent H_2_O_2_-induced cellular senescence (by decreasing p16^INK4a^, p21^WAF1^ and p53 expression and SA-β-Gal activity) in rat NP mesenchymal stem cells in vitro via the SIRT1/PGC-1α pathway [62].

### 5.16. Polydatin

Polydatin (3,4,5-trihydroxystilbene-3-beta-monoglucoside) is a naturally occurring potent stilbenoid polyphenol compound and a resveratrol derivative (resveratrol 3-O-β-D-glucoside) [200,201,202,203]. It is primarily isolated from the rhizome, dry roots and stems of the herb *Polygonum cuspidatum* and is a bioactive component of thuja, while several members of the Leguminosae, Liliaceae and Vitaceae botanical families may be its sources [200,202,204]. Polydatin is also abundant in grapes, peanuts, cocoa-containing products and red wine [63,204]. Being its natural precursor, polydatin shares health-promoting properties with resveratrol but has a more favorable pharmacological profile due to its improved clearance rate and bioavailability [204,205,206,207,208]. The presence of a benzene ring with a hydroxyl group in polydatin’s chemical structure (Figure 1) provides the molecule with a powerful free radical scavenging ability and anti-oxidant potential, while its glucose moiety renders polydatin more soluble in water, allowing it to readily exert its beneficial effects on cells and tissues [202,203]. Besides its anti-oxidant activity, numerous other biological activities have been suggested for polydatin with promising therapeutic and protective effects, including anti-inflammatory, anti-apoptotic, anti-aging, anti-cancer, anti-microbial, anti-diabetic, cardioprotective, gastroprotective, hepatoprotective and neuroprotective properties [200,201]. Thus, polydatin has been shown to be effective against several pathological conditions, such as tumors, cardiovascular diseases, ischemic reperfusion injuries, diabetes, neurodegenerative diseases, atherosclerosis, hepatic disorders, liver diseases, diseases of the respiratory system, disorders of the renal system, gastrointestinal diseases, infectious diseases, rheumatoid diseases, metabolic diseases and skeletal disorders [200,204]. Among the signaling molecules modulated by polydatin, Nrf2, NF-κΒ, mTOR, STAT17, SIRT1, PGC-1α, p38 MAPK, NLRP3 inflammasome and p53 have been reported [202,204,209].

Polydatin has been previously shown to inhibit IL-1β-mediated inflammatory and ECM catabolic phenotype in human and rat chondrocytes in vitro through the implication of the Nrf2 and NF-κB-Wnt/β-catenin pathways, respectively and to attenuate cartilage degradation in an anterior cruciate ligament transection-induced rat and a surgical DMM mouse OA models in vivo [210,211]. In addition, polydatin-induced up-regulation of Parkin and Nrf2 has been shown to protect human endplate chondrocytes against H_2_O_2_-induced mitochondrial membrane potential disruption, ROS production and apoptosis in vitro and to ameliorate cartilaginous endplate and IVD degeneration in a puncture-induced IDD rat model in vivo [212]. In the IVD, polydatin has been shown to decrease the level of senescence in TNF-α-treated rat NP IVD cells in vitro, as indicated by the reduction of positive SA-β-Gal staining and expression levels of the senescence markers p53 and p16^INK4a^ [63]. Moreover, treatment with the compound restored mitochondrial function and reduced MMP-3, MMP-13 and ADAMTS-4 expression, along with the increase in aggrecan and type II collagen levels [63]. All anti-senescence, anti-oxidant and anti-catabolic effects of polydatin on TNF-α-stimulated NP cells were moderated by siRNA-mediated Nrf2 knocking-down, implicating the activation of the Nrf2/HO-1 pathways in the mode of polydatin’s protective action [63]. Most importantly, polydatin’s senotherapeutic activity was confirmed in vivo because its oral administration suppressed senescence and ameliorated IDD development in a rat tail IVD-punctured model (as shown by the decrease of p53 and p16^INK4a^ expression and the positive alcian blue staining in the IVD tissues, the recovery of the MRI signal intensity and the lower Pfirmann grades) [63].

### 5.17. Proanthocyanidins

Proanthocyanidins are naturally occurring polyphenolic compounds present in the flowers, nuts, bark and seeds of a wide variety of plants in edible berries (i.e., lingonberry, cranberry, mulberry, black elderberry, black chokeberry, black currant, blueberry) and in almost all fruits (i.e., persimmon, banana, medlar, plum, apricot, walnut, silverberry, pomegranate). They are also found in Chinese quince (*Pseudocydonia sinensis* of the Rosaceae family), carob beans (*Ceratonia siliqua* of the Fabaceae family), rose hips and cocoa [213,214]. Proanthocyanidins (Figure 1), known as condensed tannins, are oligomers or polymers of monomeric flavan-3-ols, including catechin and epicatechin as building blocks and are produced in the phenylpropanoid biosynthetic pathway. Naringenin is the central intermediate that produces flavonoids and, ultimately, proanthocyanidins following a series of oxidation, hydroxylation, reduction and deoxygenation reactions [214]. Many studies have shown several biological activities of proanthocyanidins, including anti-oxidant, anti-apoptosis, anti-aging, anti-bacterial, anti-viral, anti-carcinogenic, anti-inflammatory, anti-allergic, anti-lipid peroxidation, anti-platelet aggregation, anti-obesity, anti-diabetic, immunomodulatory, cardioprotective and neuroprotective [214,215,216,217,218]. These activities are mediated by signaling molecules and pathways such as NF-ĸB, MAPK, PI3K/Akt, SIRT1, NLRP3 inflammasome, VEGF, MMPs, cell cycle regulators, etc., and have found application in the prevention or treatment of numerous (mainly age-associated) diseases and pathological conditions (i.e., cardiovascular diseases, neuropathy, retinopathy, nephropathy, cancer, urinary and dental infections, photodamage, blood circulation problems, ocular disorders, musculoskeletal disorders) [213,214].

Cocoa polyphenols extract (with an approx. 60% content in proanthocyanidins) has been shown to attenuate mitochondrial dysfunction and senescence through SIRT1, SIRT3, FOXO3 and p53 in H_2_O_2_-treated auditory cells [219], while grape seed proanthocyanidin extract has been shown to alleviate mitochondrial damage and to significantly decrease p16^INK4a^ and p21^WAF1^, as well as SASP levels in H_2_O_2_-induced senescent retinal pigment epithelium cells through the nicotinamide phosphoribosyltransferase (NAMPT)/SIRT1/NLRP3 pathway [220]. A hydroxytyrosol/procyanidins extract of olive and grape seed (metabolized in proanthocyanidins derivatives in rabbit serum, as revealed by mass spectrometry) had anti-inflammatory and chondroprotective effects in IL-1β-stimulated rabbit articular chondrocytes in vitro and exerted anti-OA effects in two animal models of post-traumatic OA in mice and rabbits [221]. Grape seed proanthocyanidin extract was found to be antinociceptive and protective against joint injury in a monosodium iodoacetate (MIA)-induced OA rat model [222]. In accordance with their reported anti-senescence role in other tissues and their chondroprotective effect, treatment with proanthocyanidins has been shown to confine IL-1β-induced senescence in rat NP IVD cells in vitro by attenuating p53 phosphorylation, p21^WAF1^ and p16^INK4a^ expression and SA-β-Gal activity via the PI3K/Akt pathway [64].

### 5.18. Quercetin

Quercetin (3,3′,4′,5,7-pentahydroxyavone) is one of the most important members of the flavonoid family, present in fruits (i.e., apples, red grapes, dark cherries and berries, such as blueberries and cranberries), vegetables (i.e., broccoli, onions) and other edible plants and plant-derived products, including seeds, grain, buckwheat, nuts, flowers, olive oil, green tea and red wine. Quercetin possesses anti-oxidant, anti-inflammatory, anti-aging, anti-hypertensive, vasodilator, anti-obesity, anti-hypercholesterolemic and anti-atherosclerotic properties [223,224] and has been used for the treatment and/or prevention of various pathological conditions and degenerative diseases, such as hypertension, hyperlipemia, chronic prostatitis syndromes, neurodegenerative disorders, cardiovascular diseases and OA [223,225,226,227,228,229,230,231,232]. The biological activities of quercetin may be attributed to the molecule’s chemical structure (Figure 1), consisting of three rings with two aromatic centers and a central oxygenated heterocyclic ring that provide several reactive sites (with the two hydroxyl groups of the catechol group in the B ring being the most important ones). Quercetin is insoluble in cold water [233]. The compound exerts its protective effects by interacting with cyclooxygenases, lipoxygenases and MMPs and via the modulation of several other signaling molecules, including MAPKs, Nrf2, PI3K/Akt, SIRT1, AMPK, etc. [224,227,229,230,232,234,235] Furthermore, quercetin has been reported to possess an established selective senolytic activity in several cell types and tissues, as shown using in vitro and in vivo models. For example, quercetin induced apoptosis in human senescent endothelial cells more effectively than human senescent preadipocytes [224,236], while it reduced senescence markers and improved kidney functions in high-fat diet-fed mice [237]. Additionally, a quercetin/dasatinib cocktail reduced the number of senescent cells and delayed aging in mice [224], which renders it a very popular combination in anti-senescence treatments and a promising prospect in clinical use as a therapeutic strategy for the improvement of physical condition and lifespan [238].

In the IVD, quercetin treatment protected rat NP IVD cells against apoptosis and prevented ECM degeneration induced by TBHP via the SIRT1 and p38 MAPK pathways in vitro and alleviated the progression of IDD in a needle-puncture rat model [227,239]. More specifically, regarding its anti-senescence effect, quercetin decreased p16^INK4a^, p21^WAF1^ and SASP molecules (MMP-3, MMP-13, IL-6 and IL-8) expression, as well as SA-β-Gal activity in IL-1β-treated human NP IVD cells via NF-kB signaling through Nrf2 in vitro and ameliorated the IDD process in a puncture-induced rat IDD model [66]. Novais et al. showed that treatment with a quercetin and dasatinib combination prevented age-dependent IDD progression in mice, based on the significant decrease in the senescence markers p16^INK4a^, p19^ARF^ and the SASP molecules IL-6 and MMP-13 and the amelioration of cell viability, phenotype and ECM content [65]. Furthermore, quercetin has been shown to reduce oxidative stress-induced senescence in NP-derived mesenchymal stem cells from rat tails in vitro, as shown by the recovery of cells’ proliferative potential, the decrease of TBHP-induced ROS accumulation and the reduction of the levels of senescence indicators (SA-β-Gal staining, p16^INK4a^, p21^WAF1^ and p53) and SASP factors (IL-1β, IL-6, and MMP-13) via the miR-34a/SIRT1 signaling pathway [67]. Based on X-ray and histological data, the protective role of quercetin was confirmed in vivo using a puncture-induced rat IDD model [67].

### 5.19. Resveratrol

The naturally occurring nonflavonoid polyphenol resveratrol (3,5,4′-trihydroxy-trans-stilbene) (Figure 1) is a phytoalexin, produced as a stress response in plants for their protection against fungal infections [240,241]. It can be isolated from the roots of the herbaceous perennial plant *Polygonum cuspidatum* of the knotweed and buckwheat family Polygonaceae, but its presence has also been documented in trees (i.e., eucalyptus and spruce), in flowering plants (i.e., the lily species *Veratrum grandiflorum* and *Veratrum formosanum*), in peanuts and groundnuts and in grapevines, grapes and red wine [242,243,244]. In nature, resveratrol can be found in cis- and trans-isomers, with the latter forms being the more biologically active due to the lower steric hindrance of their side chains [244]. Resveratrol’s health benefits have long been recognized because numerous studies have shown its protective effects against various diseases, such as cancer, ischemic disease, neurodegenerative diseases, cardiovascular diseases, diabetes and OA [240,242,244], as well as its ability to increase lower organisms’ lifespan and to generally improve the health of mammals [245]. The wide protective effect of resveratrol on different cell types is mediated by several signaling pathways and molecules, including NF-κB, MAPK, COX-2, AMPK and p53, that regulate its anti-inflammatory, anti-aging, anti-cancer and cartilage-protective properties [246,247].

Specifically for the IVD tissue, resveratrol has been shown to exert anti-apoptotic, anti-oxidant, anti-inflammatory, anti-catabolic and anabolic activities in vitro and using animal models through multiple signal transduction pathways towards IVD regeneration [244]. Resveratrol partly attenuated TNF-α-induced apoptosis, decreased ROS levels and increased SOD activity in rat AF IVD cells in vitro [240]. It also improved cell proliferation, inhibited apoptosis and cell cycle arrest and increased HSP90, N-cadherin, type II collagen and aggrecan expression levels in a human NP cell line by blocking the IL-6/JAK/STAT3 pathway [248]. Resveratrol has been shown to inhibit TNF-α- and IL-1β-mediated apoptosis by regulating the PI3K/Akt pathway and IL-1β-induced increase of pro-inflammatory cytokines in rat and human NP IVD cells [249,250,251]. Alone or in combination with 17β-estradiol has been shown to attenuate TNF-α-induced MMP-3 expression via the AMPK/SIRT1 pathway or to prevent IL-1β induced apoptosis in human NP IVD cells via the PI3K/Akt/mTOR and the PI3K/Akt/glycogen synthase kinase-3β (GSK-3β) pathway, respectively [252,253] and to protect them against H_2_O_2_-induced mitochondrial dysfunction and cytotoxicity through autophagy activation [254]. Additionally, it blocked basic fibroblast growth factor (bFGF)- or IL-1-induced MMP-13 and ADAMTS-4 up-regulation and significantly increased proteoglycan accumulation in bovine IVD cells in vitro [255]. Moreover, the compound restored NP matrix content ex vivo (as shown by increased alcian blue staining intensity and aggrecan and type II collagen expression) with the participation of the PI3K/Akt pathway in a porcine disc organ culture under high-magnitude mechanical compression [256], while resveratrol treatment in vivo decreased p16^INK4a^ expression and protected against puncture-induced IDD in the coccygeal discs of a mouse model, promoted features of regeneration in a rabbit needle puncture-induced IDD model and showed analgetic potential in a rat model of radiculopathy by the application of the NP tissue to the dorsal root ganglion [98,249,254,257,258].

When focusing on IVD senescence regulation, resveratrol has been reported to partly reverse increased SA-β-Gal activity and ROS content, decreased cell proliferation and cell cycle delay, up-regulated expression of the senescence markers p16^INK4a^ and p53 and the ECM-degrading enzymes MMP-3, MMP-13 and ADAMTS-4 and down-regulated expression of the ECM components aggrecan and type II collagen in TNF-α- and IL-1β-treated rat NP cells in vitro [69]. Given that diabetes mellitus is a potential etiological factor of IDD, the effect of high-glucose on senescence induction in NP cells was investigated [70]. High glucose indeed increased positive SA-β-Gal staining, G0/G1 phase cell cycle fraction and p16^INK4a^ and p53 expression in rat NP cells in vitro, which were all alleviated by resveratrol through activating the ROS-mediated PI3K/Akt pathway [70]. Moreover, resveratrol partly attenuated mechanical overloading-induced senescence in rat NP cells by regulating the ROS/NF-κB pathway [71]. Finally, resveratrol treatment increased cell proliferation and type II collagen expression and decreased ADAMTS-5, MMP-13, p21^WAF1^ and p16^INK4a^ levels in H_2_O_2_-induced senescent human NP IVD cells, all counteracted by siRNA-mediated SIRT1 knocking-down [68].

### 5.20. o-Vanillin

o-Vanillin (2-hydroxy-3-methoxybenzaldehyde) (Figure 1) is an isomer of the well-known food supplement vanillin and the principal metabolite of curcumin [13,259], showing though higher specificity and better bioavailability than the latter [72]. The putative therapeutic potential of o-vanillin has been studied in several pathologies, including cancer and acute kidney injury [259,260].

o-Vanillin has been shown to possess a clear senolytic activity in the IVD, efficiently killing senescent AF and NP cells [50]. More specifically, o-vanillin treatment resulted in the decrease of senescent cells, as revealed by p16^INK4a^ staining, and the increased number of caspase-3-positive (apoptotic) cells, accompanied by an increase in the cells’ proliferative potential (Ki-67-positive cells). Double-immunofluorescence staining of p16^INK4a^ with either caspase-3 or Ki-67 markers elucidated that cells driven to apoptosis were exclusively senescent cells, while the remaining cells were those that retained the ability to proliferate. Moreover, o-vanillin promoted metabolic activity and apoptosis in cells from degenerate IVDs and not from non-mildly-degenerate IVDs. In addition, o-vanillin resulted in increased type II collagen and proteoglycan levels, decreased levels of the SASP inflammatory cytokines IL-6 and -8 and down-regulation of the proteases MMP-3 and -13. o-Vanillin-conferred protective effects were shown to be mediated by the Nrf2 and NF-κB signaling pathways [50]. In their follow-up integrated study, Cherif et al. demonstrated a senomorphic function of o-vanillin in the IVD, in addition to its previously shown senolytic activity, using monolayer IVD cell cultures, pellet cell cultures and ex vivo human IVD cultures along with state-of-the-art in silico and experimental approaches, including a gene expression study of a pre-specified set of apoptotic and senescence-genes, a human cytokine antibody array, a Luminex multiplex assay and intact IVD tissue immunohistochemistry [72]. In this study, it was shown that o-vanillin significantly modulated the expression of apoptotic and cell cycle-regulating genes [i.e., Bcl-2, cyclin-dependent kinase (CDK) 6, CDK2C, CDK2D, cell division cycle (CDC) 25c, CDK2A, cyclin A2, cyclin D1 and cyclin B1), while decreasing the overall inflammatory IVD environment by reducing SASP factors, such as IFN-γ, IL-6, CC chemokine ligand (CCL) 24, IL-7, IL-8, CCL7, CCL26, CXC chemokine ligand (CXCL) 1, CXCL5, CXCL6, CXCL10 and VEGF-A. These findings support a more potent, exhaustive senotherapeutic activity of o-vanillin, resulting from the concomitant selective elimination of senescent cells (senolysis) and suppression of inflammatory agents (semomorphic activity) that may help healthy cells to grow and retard or prevent SASP-induced bystander activation of senescence [50,72]. In accordance with the reported association between increased TLR expression and increased degree of disc degeneration and pain [261], double staining for p16^INK4a^ and TLR-2 revealed their co-localization in human TLR-2/6 activation-induced senescent IVD cells from non-degenerate and degenerated tissues [73]. o-Vanillin administration has been shown to result in the down-regulation of p16^INK4a^, TLR-2 and SASP factors [73].

Human mesenchymal stem cell (hMSC)- and extracellular vesicle (EV)-therapy is a promising treatment for discogenic LBP, the effectiveness of which may be jeopardized by senescence-associated reduction of self-renewal and SASP-induced disruption of tissue homeostasis. Thus, improvement of regenerative approaches may rely on the reduction of senescence. o-Vanillin has been shown to enhance hMSC differentiation and improve IVD cells’ phenotype by increasing proteoglycan synthesis, decreasing the number of p16^INK4a^-positive senescent cells and decreasing the release of IL-6 and -8 in co-cultures of IVD cells and hMSCs [74]. In addition, it significantly increased EV release and/or uptake by hMSCs and IVD cells, while conditioned media of o-vanillin-treated cells stimulated the up-regulation of IVD markers in both cell types [74].

## 6. Plant-Derived Metabolites with a Potential Senotherapeutic Role against IDD

**Figure 2:**
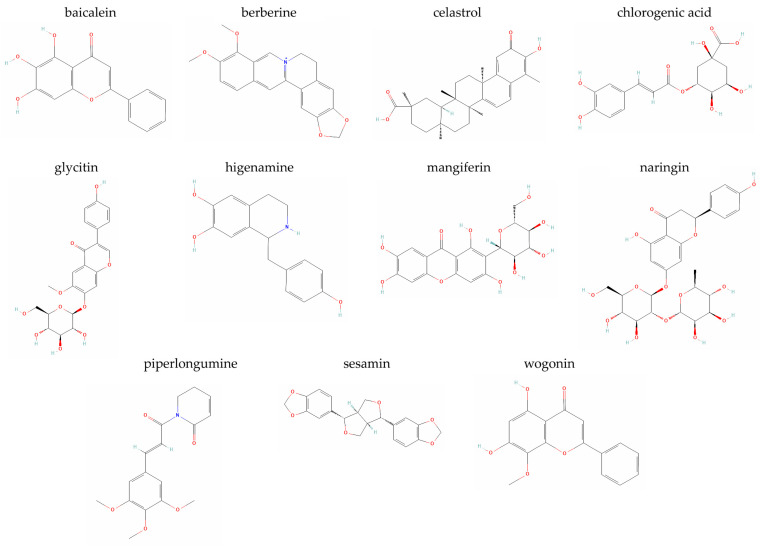
Chemical structures of plant-derived metabolites with potential senotherapeutic activity in the intervertebral disc, retrieved from the public chemical database PubChem (https://pubchem.ncbi.nlm.nih.gov).

Apart from the above-mentioned natural compounds with a reported anti-senescence mode of action in the IVD, there are also other plant-derived metabolites (Figure 2) that have been demonstrated to exert beneficial effects in the IVD or the closely related cartilage in vitro or in vivo, as well (Table 2). Even though in these cases the conferred protective effects have not been tested for their association with a senotherapeutic action in the IVD thus far, anti-oxidant, anti-inflammatory and anti-catabolic properties have been described for them, rendering them ideal candidates in the pursuit of novel senomorphics for the particular tissue. For example, (i) *baicalein* has been shown to inhibit IL-1β-induced inflammatory response and ECM degradation in rat NP IVD cells in vitro and to attenuate IDD in vivo [262], as well as to alleviate TNF-α-induced MMP-2 and -9 up-regulation in human NP IVD cells [263]; (ii) *berberine* has been shown to inhibit the TBHP-induced production of ECM-degrading enzymes in rat NP IVD cells in vitro and to prevent the development of IDD in a needle puncture-induced rat model [264]; (iii) *celastrol* has been reported to suppress IL-1β-stimulated up-regulation of COX-2, IL-6, PEG2 and MMP-13 in rat chondrocytes in vitro and to delay the progression of cartilage damage in an OA rat model [265], to play an anti-inflammatory role in rheumatoid arthritis and to suppress the expression of MMP-1, -3 and -13, COX-2, iNOS and HSP90β in primary human OA chondrocytes [266,267], to reduce the production of inflammatory mediators preventing the destruction of articular cartilage after intra-articular injection in a MIA-induced knee OA rat model [268], to reduce IL-1β-induced ECM catabolism, oxidative stress and inflammation in human NP IVD cells and to attenuate rat IDD in vivo [269] and most importantly to be able to target multiple genes involved in cellular senescence in OA based on a comparative transcriptomics and network pharmacology analysis [270]; (iv) *chlorogenic acid* has been reported to mitigate cartilaginous endplate degeneration and to postpone IDD development in a lumbar spine instability IDD mouse model [271]; (v) *glycitin* has been reported to antagonize TNF-α-induced cartilage degeneration and inflammation in primary mouse chondrocytes and TNF-α-induced inflammatory response and ECM catabolism in human NP IVD cells in vitro, while its intraperitoneal administration rescued tissue destruction in an anterior cruciate ligament transection OA mouse model and a puncture-induced IDD rat model [272,273]; (vi) *higenamine* has been shown to attenuate IL-1β-induced elevation of ROS levels, inflammatory mediators and catabolic markers in human NP IVD cells [274,275]; (vii) *mangiferin* treatment has been shown to decrease proliferation, migration and secretion of inflammatory cytokines and MMPs and to promote apoptosis of fibroblast-like synoviocytes in vitro, as well as to alleviate arthritis index, to down-regulate the production of inflammatory mediators and ECM-degrading enzymes and to ameliorate oxidative stress in the plasma and the synovial tissue in an adjuvant-induced arthritis rat model [276]. Furthermore, mangiferin has been reported to attenuate inflammatory responses, to reverse the loss of ECM components, to reduce ROS production and to ameliorate mitochondrial damage in TNF-α-stimulated human NP IVD cells and cultured mouse IVD tissues, as well as to protect against IDD in a puncture-induced rat IDD model [277]; (viii) *naringin* has been shown to inhibit cyclic stretch-induced mitochondrial membrane potential depolarization and oxidative stress in rat AF IVD cells in vitro and to marginally decrease Pfirrmann MRI grades in a static and dynamic imbalance-induced IDD rat model [278], to protect human NP IVD cells against TNF-α- and IL-1β-induced inflammation, oxidative stress and loss of cellular homeostasis [279,280] and to increase the expression of type II collagen and aggrecan in human NP IVD cells isolated from degenerated IVD specimens [281]; (ix) *piperlongumine*—with an established senolytic activity in ionizing radiation-induced senescent normal human WI-38 fibroblasts [150] and having emerged as the top-runner in a computational screening for natural senotherapeutic repurposing candidates that mimic dasatinib based on gene expression data [282]—has been reported to rescue IL1β-induced elevated levels of oxidative stress in cartilage explants and to reduce the expression of major inflammatory markers in a goat ex vivo OA model [283]; (x) *sesamin* has been found to protect against IDD based on a network pharmacology analysis [284] and to inhibit LPS-induced inflammation and ECM catabolism in the rat IVD in vitro and ex vivo [285], while its intradiscal injection in a rat tail disc has been shown to protect from lesion-induced IDD in vivo [286]; (xi) *wogonin* has been reported to suppress inflammatory mediators and ECM-degrading enzymes and to up-regulate type II collagen in IL-1β-stimulated rat NP IVD cells and to ameliorate IDD in an in vivo rat caudal vertebrae needle-stab model [287].

## 7. Perspectives, Challenges and Practical Concerns

Here we presented a number of plant-derived compounds that have been reported to possess a senotherapeutic activity or that show a senotherapeutic potential and could be exploited as nutraceuticals or even pharmaceuticals for the prevention and treatment of IVD degeneration and aging. However, open questions and issues still remain to be addressed to accomplish the highest degree of specificity, bioavailability and efficacy of these natural metabolites for the IVD tissue.

Given that cellular senescence is a multi-parametric physiological state, with no single universal and specific marker for its unequivocal identification described so far, a thorough characterization of senescent cells is needed based on the concurrent expression of many senescence-associated phenotypic, genetic and molecular traits. Otherwise, transient stress responses to short-term treatments could be mistakenly described as senescent phenotypes. Thus, to avoid erroneous attribution of senomorphic traits to compounds that just have an anti-oxidant, anti-inflammatory or anti-catabolic action not necessarily associated with senescence, the establishment of a solid senescent in vitro, ex vivo or in vivo IVD model needs to be the first step in any study assessing potential senotherapeutic activities. In this direction, the recently described two-phase algorithmic assessment to quantify various senescence-associated parameters in the same specimen could be employed, combining the measurement of lysosomal and proliferative features, the expression of general senescence-associated genes and the measurement of SASP factors’ levels [291]. In addition, even though immortalized cells have been used in some of the afore-mentioned works [59,144], the use of normal (preferably primary) IVD cell cultures is required for this type of study.

Moreover, with few exceptions, most studies attributing a senotherapeutic potential to a compound were based on the general characterization of an ameliorated senescent phenotype, not sufficient to clarify if this beneficial effect is the result of the selective elimination of senescent cells (senolysis) or the suppression of their SASP (senomorphism). Co-staining for senescence and apoptosis markers or comparative viability curves of early-passage (young) and senescent cells in the presence of the compound of interest would provide direct evidence on the prevalence or not of senescence-specific cytotoxicity.

Furthermore, most studies on natural senotherapeutic compounds for IVD have focused on NP cells, even though damage to the AF and the cartilaginous endplate has also been linked to IDD [292,293]. It seems rational that higher efficacy of a particular treatment at the tissue level in vivo may be achieved if its active ingredients positively affect AF and NP IVD cells in addition to the contiguous non-IVD cells. Indeed, the beneficial effect of quercetin/dasatinib administration against IDD was demonstrated to be a result of the drug combination not only on IVD cells per se but on the adjacent vascular endothelial cells, as well. Given that aging is accompanied by the gradual decrease of microvessels’ number under the bony endplate due to cartilaginous endplate calcification that worsens nutrient availability of NP cells, vascular endothelial cells’ senescence could pave the way towards IDD [294,295]. Besides, an inevitable interrelationship between IVD cells and vascular endothelial cells has been reported [296], while endplate degeneration and blockade of nutrient supply from the vessels have been shown to precede NP IVD degeneration [297]. Treatment with the combination of quercetin and dasatinib reduced the number of senescent vascular endothelial cells (as shown by the lower SA-β-Gal activity and p16^INK4a^ and p21^WAF1^ expression) in the marrow space of the bony endplate in aged mice, which was accompanied by an increase in the number of both endothelial and osteoblast cells, a higher expression of angiogenic markers in the sub-endplate region and ultimately an improved histological evaluation of IDD [289].

Combinations of a natural senotherapeutic with other natural or synthetic senolytic or senomorphic compounds to simultaneously target multiple cell types and anti-senescence pathways should be examined, as they could prove more advantageous and efficient than using a single senotherapeutic agent in the prevention/therapy against IVD degeneration and aging. The quercetin and dasatinib combination selectively targeted a broader range of senescent cell types than either agent alone [224]. Moreover, compared with the single treatments, the combination of o-vanillin and RG-7112 significantly reduced the amount of senescent IVD cells, pro-inflammatory cytokines and neurotrophic factors [4].

The protective effects of natural compounds on IDD have been validated in vivo, mainly in rodents and, more specifically, primarily using the tail puncture model that is far from simulating human IDD. Thus, new and suitable animal models need to be progressively established so as to allow the design of more efficient clinical studies in the future.

Finally, given the avascular nature and the special physicochemical environment of the IVD, it is challenging to select the most appropriate mode of administration (oral, intravenous, intraperitoneal) for each senotherapeutic based on its solubility, half-life and clearance rate in vivo because local intradiscal injections could not be an option as they increase the risk of IDD [298]. Furthermore, the bioavailability and distribution of potential agents for the treatment of IDD in the plasma, as well as successful delivery into the IVD tissue, should always be inspected. We have recently developed a solid phase extraction liquid chromatography-tandem mass spectrometry (SPE-LC-MS/MS) analytical method, showing that injection of the bisphosphonate zoledronic acid in a rabbit model resulted in rapid accumulation and quick clearance in the plasma and the skin, but lower and delayed accumulation in the AF and no zoledronic acid detection in the NP of the IVD [299]. The bioavailability of fisetin has been studied following intravenous and oral administration, showing that free fisetin’s serum levels decline rapidly within the first few hours [5]. A specific and sensitive ultra-performance liquid chromatography-tandem mass spectrometry (UPLC-MS/MS) method has been developed for the identification and quantification of myricetin in the rat plasma after oral and intravenous administrations, which indicated that myricetin would be poorly absorbed in the gastrointestinal tract, possibly due to its low water solubility, low stability in the gastrointestinal fluid and rapid biotransformation [300]. A validated reversed-phase HPLC method was developed to determine the in vitro penetration and in vivo distribution of honokiol into the IVD, suggesting that the penetration of honokiol into the IVD is sufficient to achieve a therapeutic concentration after honokiol administration [301].

Improvement of pharmacokinetics properties could be attained by chemical modification, providing new analogs or derivatives of the most promising candidates with reported senotherapeutic activity. In addition, to achieve a targeted delivery, a high degree of bioavailability and a controlled drug release, the use of biomaterial-based delivery scaffolds and systems has been suggested. Microspheres, for example, have been employed for the local delivery of several therapeutic factors for the treatment of various diseases based on their good biocompatibility, degradability, mechanical properties and injectability combined with satisfactory drug encapsulation and delivery efficiencies [298,302,303]. A novel and simple way for packaging multiple functions into a single delivery platform by using vanillin and TGF-β3 in gelatin methacrylate (GelMA) microspheres has been proposed for IDD treatment [290]. Notably, the functionalized microspheres indeed retained desirable pharmacological values of vanillin and improved release kinetics of TGF-β3, as shown in NP IVD cells in vitro and in a rat IDD model in vivo [290]. Moreover, given the rapid metabolism and poor bioavailability of curcumin due to its high hydrophobicity, low solubility and instability in water [119], a composite scaffold with curcumin encapsulated in solid lipid nanoparticles (SLNs) and mixed with GelMA hydrogel was tested for its efficacy to treat IDD. Indeed, curcumin/SLNs inhibited the expression of the inflammatory factors TNF-α and IL-6 in IL-1β-treated rat NP IVD cells in vitro, while the GelMA scaffold successfully promoted the restoration of collagen type II and aggrecan expression levels in a rat IDD model in vivo [120]. Kaempferol is soluble in organic solvents and slightly soluble in water, as well. An injectable kaempferol-loaded fibrin glue that was developed securing good injectability, biocompatibility and sustained slow release was shown to reduce the inflammatory response and ECM breakdown in LPS-stimulated rat NP IVD cells in vitro and to improve needle puncture-induced IDD in a rat model [288]. Finally, due to its low solubility, hollow mesoporous silica nanoparticles were used to deliver celastrol via intra-articular injection in a MIA-induced knee OA rat model, and the results showed that such a system can improve the bioavailability of celastrol [268].

## 8. Concluding Remarks

Many natural compounds have been reported to possess a senotherapeutic potential against IDD, and although naturally derived senotherapeutics may be less potent than synthetic chemicals, they seem to be an advantageous choice for translation into clinical settings due to their low toxicity and absence of adverse side-effects. Based on the above presented data, we could propose a holistic and multi-disciplinary strategy for the discovery and evaluation of competent plant-derived senotherapeutic compounds that could secure a better design of future clinical trials against IDD: (i) exhaustive characterization of the senescent phenotype induced using different protocols for senescence induction in appropriately selected in vitro, ex vivo and in vivo IVD models needs to precede any screen for novel senotherapeutics; (ii) design of adequate experimental setups is required to ascertain selective activity of discovered/identified compounds on senescent cells by performing simultaneous comparative analyses with early-passage cells; (iii) emerging senotherapeutic compounds could be tested in all IVD cell types (AF, NP, cartilaginous endplate cells), as well as in cells of tissues adjacent and interacting with the disc to achieve maximal efficacy; (iv) assessment of the pharmacokinetics properties of the compounds is necessary to evaluate their ability to reach their destination, especially given the avascular nature of the IVD; (v) appropriate chemical modifications, biomaterial-based scaffolds and combinations of compounds could be attempted to refine and improve delivery and efficacy. Taking into account the multiparametric nature of cellular senescence, the peculiar physicochemical environment of the IVD and the structural complexity of the tissue, this approach seems required for the selection of the most promising candidates—holding both a high degree of bioactivity and desirable pharmacokinetics properties—that could be used alone or in combinations in therapeutic strategies with the maximal possible health benefit for the prevention and/or treatment of IVD degeneration and aging.

## Figures and Tables

**Table 1:** Plant-derived metabolites with a reported senotherapeutic activity in the intervertebral disc and proposed implicated signaling pathways in their action.

Plant-Derived Compound	Senotherapeutic Activity	Implicated Signaling Pathway(s)	Reference(s)
Apigenin	Senomorphic	AMPK-mTOR-TFEB	[45]
Butein	Senomorphic	Sirt1-p53	[46]
p-Coumaric acid	Senomorphic	ND *	[47]
Curcumin	Senomorphic	AMPK-mTOR-ULK1	[48]
		p70/S6K, Akt-LC3-II-SQSTM1/p62	[49]
	Senolytic	JNK	[50]
Dehydrocostus lactone	Senomorphic	STING-TBK1-NF-κB, MAPK	[51]
20-Deoxyingenol	Senomorphic	TFEB-autophagy/lysosome pathway	[52]
Eupatilin	Senomorphic	MAPK-NF-κB	[53]
Evodiamine	Putative senomorphic	Nrf2-HO-1, MAPK	[54]
Fisetin	Senomorphic	ND	[43]
Honokiol	Senomorphic	AMPK-PGC-1α-SIRT3	[55]
Kaempferol	Senomorphic (network pharmacology analysis/in vitro)	MAPK	[56]
		ND	[57]
Kinsenoside	Senomorphic	Akt-ERK1/2-Nrf2	[58]
Luteolin	Senomorphic	SIRT6-NF-κB	[59]
Morroniside	Senomorphic	ROS-Hippo-Mst1/2 and Lats1/2-YAP/TAZ-p53	[60]
Myricetin	Senomorphic	SERPINE1	[61]
		SIRT1-PGC-1α	[62]
Polydatin	Senomorphic	Nrf2-HO-1	[63]
Proanthocyanidins	Senomorphic	PI3K-Akt	[64]
Quercetin	Senolytic (quercetin/dasatinib combination)	ND	[65]
	Senomorphic	Nrf2-NF-κB	[66]
		miR-34a-5p-SIRT1	[67]
Resveratrol	Senomorphic	SIRT1	[68]
		ND	[69]
		ROS-PI3K-Akt	[70]
		ROS-NF-κB	[71]
o-Vanillin	Senolytic (o-vanillin/RG-7112 combination)	ND	[4]
	Senolytic/senomorphic	JNK, Nrf2, NF-κB	[50]
		ND	[72]
		TLR-2	[73]
		ND	[74]

* ND: Not determined.

**Table 2:** Plant-derived metabolites with a reported protective effect on IDD using various experimental settings, cell and animal models.

Plant-Derived Compound	Setting	Cell/Animal Model	Reference(s)
Apigenin	In vitro	Rat NP IVD cells	[45,87]
	In vivo	Puncture-induced rat IDD model	
Baicalein	In vitro	Rat and human NP IVD cells	[262,263]
	In vivo	Puncture-induced rat IDD model	[262]
Berberine	In vitro	Rat NP IVD cells	[264]
	In vivo	Puncture-induced rat IDD model	
Butein	In vitro	Rat NP IVD cells	[46]
	In vivo	Streptozotocin-/puncture-induced rat diabetes and IDD model	
Celastrol	In vitro	Human NP IVD cells	[269]
	In vivo	Puncture-induced rat IDD model	
Chlorogenic acid	In vivo	Lumbar spine instability-induced IDD mouse model	[271]
p-Coumaric acid	In vitro	Human NP IVD cells	[47]
		Human degenerated IVD cells	[110]
Curcumin	In vitro	Human IVD cells	[50,115]
		Rat NP IVD cells (curcumin/SLNs mixed with GelMA hydrogel)	[120]
		Human NP IVD cells	[48,49]
		Human cartilaginous endplate cells	[121]
	In vivo	Puncture-induced rat IDD model	[48,123]
		Surgically-induced lumbar rat IDD model	[122]
Dehydrocostus lactone	In vitro	Rat NP IVD cells	[51]
	In vivo	Spinal instability-induced mouse model	
20-Deoxyingenol	In vitro	Rat NP IVD cells	[52]
	In vivo	Puncture-induced rat IDD model	
Eupatilin	In vitro	Rat NP IVD cells	[53]
	In vivo	Puncture-induced caudal rat IDD model	
Evodiamine	In vitro	Immortalized human NP IVD cells	[144]
		Rat NP IVD cells	[54]
	In vivo	Puncture-induced rat IDD model	
Fisetin	In vitro	Rat NP IVD cells	[43]
		Primary rat NP mesenchymal stem cells	[156]
	In vivo	Puncture-induced rat IDD model	[43]
Glycitin	In vitro	Human NP IVD cells	[272]
	In vivo	Puncture-induced rat IDD model	
Higenamine	In vitro	Human NP IVD cells	[274,275]
Honokiol	In vitro	Rat NP IVD cells	[55,164]
	In vivo	Puncture-induced rat IDD model	
Kaempferol	Network pharmacology analysis/In vitro	Human NP IVD cells	[56]
	Network pharmacology analysis	-	[57]
	In vitro	Rat NP IVD cells (kaempferol-loaded fibrin glue)	[288]
	In vivo	Puncture-induced rat IDD model (kaempferol-loaded fibrin glue)	
Kinsenoside	In vitro	Rat NP IVD cells	[58]
	In vivo	Puncture-induced caudal rat IDD model	
Luteolin	In vitro	Immortalized human NP IVD cells	[59]
Mangiferin	In vitro	Human NP IVD cells	[277]
	Ex vivo	Cultured mouse IVD tissues	
	In vivo	Puncture-induced rat IDD model	
Morroniside	Network pharmacology analysis/In vivo	Lumbar spine instability-induced IDD rat model	[191]
	In vitro	Rat NP IVD cells	[60]
	In vivo	Lumbar spine instability-induced IDD mouse model	
Myricetin	In vitro	Human NP IVD cells	[61]
		Rat NP mesenchymal stem cells	[62]
Naringin	In vitro	Rat AF IVD cells	[278]
		Human NP IVD cells	[279,280]
		Human degenerated NP IVD cells	[281]
	In vivo	Static and dynamic imbalance-induced IDD rat model	[278]
Polydatin	In vitro	Rat NP IVD cells	[63]
		Human endplate chondrocytes	[212]
	In vivo	Puncture-induced rat IDD model	[63,212]
Proanthocyanidins	In vitro	Rat NP IVD cells	[64]
Quercetin	In vitro	Rat and human NP IVD cells	[66,227,239]
		Rat NP-derived mesenchymal stem cells	[67]
		Human umbilical vein endothelial cells (quercetin/dasatinib combination)	[289]
	In vivo	Naturally aged mice (quercetin/dasatinib combination)	[65,289]
		*Ercc1*^−/Δ^ mice (quercetin/dasatinib combination)	[224]
		Puncture-induced rat IDD model	[66,67,227,239]
Resveratrol	In vitro	Rat and human NP IVD cells	[68,69,70,71,248,249,250,251,252,253,254]
		Rat AF IVD cells	[240]
		Bovine NP IVD cells	[255]
	Ex vivo	Porcine disc organ culture	[256]
	In vivo	Puncture-induced mouse IDD model	[98]
		Puncture-induced rabbit IDD model	[254,257]
		Rat model of radiculopathy	[249,258]
Sesamin	Network pharmacology analysis/In vitro	ATDC5 cell line	[284]
	In vitro	Rat NP IVD cells	[285]
	Ex vivo	Rat lumbar IVD organ cultures	
	In vivo	Lesion-induced rat IDD model	[286]
o-Vanillin	In vitro	Human degenerated IVD cells and pellet cultures (o-vanillin/RG-7112 combination)	[4]
		Human IVD cells and pellet cell cultures	[50,72]
		Non-degenerate and degenerated human IVD cells	[73]
		Human mesenchymal stem cells	[74]
		NP IVD cells (GelMA microspheres with vanillin/TGFβ3)	[290]
	Ex vivo	Human IVD organ cultures	[72]
	In vivo	Puncture-induced rat IDD model (GelMA microspheres with vanillin/TGFβ3)	[290]
Wogonin	In vitro	Rat NP IVD cells	[287]
	In vivo	Rat caudal vertebrae needle-stab model	

## Data Availability

Not applicable.
