# Correction: Mavrogonatou, E.; Kletsas, D. Plant-Derived Senotherapeutics for the Prevention and Treatment of Intervertebral Disc Degeneration and Aging. *Metabolites* 2024, *14*, 146

**DOI:** 10.3390/metabo16070474

**Published:** 2026-07-07

**Authors:** Eleni Mavrogonatou, Dimitris Kletsas

**Affiliations:** Laboratory of Cell Proliferation and Ageing, Institute of Biosciences and Applications, National Centre for Scientific Research “Demokritos”, 15341 Athens, Greece; dkletsas@bio.demokritos.gr

The authors would like to make the following correction to their published paper [1]. The change is as follows:

References [69–71,250,256] in the original publication were retracted after the publication of our article. To ensure the quality of our work we have now removed the five retracted articles from the text, tables and reference list and deleted or revised the relevant text passages that originally cited these articles. With this correction, the order of some references has been adjusted accordingly. The authors state that the scientific conclusions are unaffected. This correction was approved by the Academic Editor. The original publication has also been updated.
